# ‘Social, innovative and smart cities are happy and resilient’: insights from the WHO EURO 2014 International Healthy Cities Conference

**DOI:** 10.1186/1476-072X-14-3

**Published:** 2015-01-14

**Authors:** Maged N Kamel Boulos, Agis D Tsouros, Arto Holopainen

**Affiliations:** The Alexander Graham Bell Centre for Digital Health, University of the Highlands and Islands, Elgin, IV30 1JJ Scotland, UK; World Health Organization Regional Office for Europe, UN City, Marmorvej 51, DK-2100 Copenhagen, Denmark; Kuopio Innovation Ltd, Viestikatu 7, FI-70600 Kuopio, Finland

**Keywords:** WHO healthy cities, Smart cities, Internet of things, Innovation, Digital inclusion, Distributed city model, Smart countryside, Social care, Well-being Europe

## Abstract

This paper provides a brief overview of, and elaborates on, some of the presentations, discussions and conclusions from Day 4 of the ‘WHO EURO 2014 International Healthy Cities Conference: Health and the City - Urban Living in the 21st Century’, held in Athens, Greece on 25 October 2014. The Internet of Things (IoT) is made of sensors and other components that connect our version of the world made of atoms, i.e., humans/our bodies, our devices, vehicles, roads, buildings, plants, animals, etc., with a mirror digital version made of bits. This enables cities and regions to be self-aware and dynamically reconfigurable in real- or near-real-time, based on changes that are continuously monitored and captured by sensors, similar to the way the internal biological systems of a living being operate and respond to their environment (homeostasis). Data collected by various IoT sensors and processed via appropriate analytics can also help predict the immediate future with reasonable accuracy, which enables better planned responses and mitigation actions. Cities and regions can thus become more adaptable and resilient in face of adversity. Furthermore, IoT can link atoms (humans) to other atoms (humans) (again via bits), resulting in the formation of ‘smart(er) communities’ that are socially connected in new ways and potentially happier. Cities, but also less urbanised regions and the countryside, could all benefit from, and harness the power of, IoT to improve the health, well-being and overall quality of life of the local populations, actively engage citizens in a smarter governance of their region, empower them to better care for one another, promote stronger social inclusion, and ensure a greener, sustainable and more enjoyable environment for all. Technology can also help reverse the ‘brain drain’ from the countryside and smaller towns to larger metropolises by making the former more attractive and connected, with better services akin to those found in larger cities. The article also discusses some ways of measuring and benchmarking the performance of smart cities and their impact on well-being. However, it should be emphasised that technology is not a panacea and that other factors are equally important in creating happier and healthier cities and regions.

**Electronic supplementary material:**

The online version of this article (doi:10.1186/1476-072X-14-3) contains supplementary material, which is available to authorized users.

## Introduction

The World health Organization Regional Office for Europe held the *‘2014 International Healthy Cities Conference: Health and the City - Urban Living in the Twenty-first Century’* in Athens, Greece, from 22 to 25 October 2014 [1, 2]. The conference marked 25 years of the European and global ‘Healthy Cities’ movement. Launched in 1987 and currently (at the time of writing) in phase VI (2014–2018), the movement is one of the most enduring and thriving international public health initiatives to date [3, 4]. The conference was an excellent forum to share visions and best solutions for cities committed to health and well-being. It was well attended by delegates from the networks of healthy municipalities, healthy communities, healthy villages, healthy districts and healthy islands and other similar WHO initiatives; representatives of all levels of government from various countries: ministers, mayors, councillors, members of regional assemblies and members of parliament; delegates from community, voluntary and non-governmental organisations; academics and professionals working for urban health in areas such as public health, urban planning, regeneration, health promotion, environmental health, social care, urban economics, primary health care, community development and equity; representatives from relevant international agencies; and staff members from all WHO regional offices and headquarters.

The last day of the conference, Day 4 (25 October 2014), focused on the theme of ‘smart and innovative cities (social, innovative and smart = happy cities)’, discussing among other topics: ‘how can we create and measure happy and resilient cities’ and the roles and challenges posed by Internet of Things (IoT) technologies in the realisation of happier cities [5]. This paper provides a brief overview of, and elaborates on, some of the presentations, discussions and conclusions from Day 4.

‘Creating self-aware and smart healthy cities’ was the title of the plenary keynote by Maged N. Kamel Boulos, Professor and Chair of Digital Health, Alexander Graham Bell Centre for Digital Health, University of the Highlands and Islands, Scotland, UK [6]. Christine McLaren, journalist, researcher and co-founder, Discourse Media, Vancouver, Canada, then spoke about the happy social city, that ‘happy’ and ‘social’ are closely tied, and the role played by urban design in creating such environments. Presentations were also delivered by Arto Holopainen, Development Director, Kuopio Innovation Ltd, Finland, on the topic of ‘Innovating for health and well-being’, drawing on the Finnish experience in this respect [7], and Carrie Exton, Policy Analyst, Organisation for Economic Co-operation and Development (OECD), on ‘Understanding well-being’ and using the OECD Better Life Index to measure it, focusing on ‘system outputs’ rather than ‘inputs’ in 11 areas of material living conditions and quality of life [8]. A round table chaired by Agis D. Tsouros, Head, Policy, Cross-cutting Programmes and Regional Director’s Special Projects, WHO EURO, followed, entitled ‘Promoting innovation – creating self-aware, smart healthy cities’ and was an excellent opportunity for the audience to contribute new insights to the discussion. Round table participants were Maged N. Kamel Boulos and Arto Holopainen.

## Creating self-aware and smart healthy cities: potential, requirements and challenges

A major theme of Day 4 was the Internet of Things (IoT) and its role in creating smart healthy cities. The IEEE IoT Technical Community defines IoT as “*a self-configuring and adaptive system consisting of networks of sensors and smart objects whose purpose is to interconnect” all “things, including every day and industrial objects, in such a way as to make them intelligent, programmable and more capable of interacting with humans*” [9]. IoT is made of sensors and other components that instrument and connect our version of the world made of atoms, i.e., our human body, our devices, vehicles, roads, buildings, plants, animals, etc., with a mirror digital version made of bits [10]. This enables cities and regions equipped with IoT technology to become self-aware of their environment, dynamically reconfigurable and adaptable in real- or near-real-time, and thus more resilient and better prepared in their response to adversity [11], based on changes that are continuously monitored and captured by sensors, similar to the way homeostasis operates in living beings. The context-aware networks and sensing infrastructure of smart homes and cities also facilitate the delivery of smarter health and social care services, opening up superior and safer active living opportunities that better meet the needs of the increasingly ageing populations in Europe and elsewhere around the world [12, 13].

In his plenary keynote on Day 4 [6] (Additional file 1; and also in [13]), Kamel Boulos offered a number of examples of IoT-driven services implemented by the Spanish city of Barcelona and by Sant Cugat (a suburb north of Barcelona) to improve the quality of life of the local populations and ensure a greener and more sustainable environment. However, Barcelona is not the only smart city in Europe that has deployed such services. Nice (France), Hamburg (Germany), Milton Keynes (UK) (see slide 30 in [6]) and many other cities across Europe [14, 15] already have similar programmes in place at various stages of development. The European Commission’s EIP-SCC (European Innovation Partnership on Smart Cities and Communities) runs an online marketplace where hundreds of smart city solution proposals, projects and developments from >300 European Region cities can be browsed [16].

### High-speed Internet (30–100 Mbps or better)

Common to all these smart cities programmes and developments is the need for superfast Internet infrastructure, functioning as the essential connectivity backbone for all IoT traffic and services (cf. Barcelona’s 500 Km fibre network [6, 13], and in UK, the government’s investment of £146 m in cable broadband in the Highlands and Islands, Scotland [17], and similar investments in South West England). A good mobile broadband (3G/4G and soon 5G - 3rd, 4th and 5th generations) coverage is often also necessary to supplement city and region-wide cable broadband and Wi-Fi (Wireless Fidelity) hotspots and support seamless uninterrupted mobile scenarios and applications in areas with no cable broadband/Wi-Fi access (e.g., intercity highways, smart countryside and distributed cities -- see below). A balanced and efficient network bandwidth management approach is also needed, paying special attention to specialised services such as critical emergency or health applications, while at the same time applying very minimal (temporary) or preferably no throttling to online content and other services intended for ordinary Internet users.

### Big data analytics

IoT sensors, including body-worn ‘quantified self’ sensors, generate big data streams. Such data are of little value without the appropriate analytics to process and make sense of them in useful ways and in a timely manner, e.g., to support making more informed decisions and/or to react or pro-act by triggering appropriate actions. Three main complementary levels of analytics are recognised. Descriptive analytics are concerned with what has happened (or is happening right now) and where it happened (or is happening). Anticipatory or predictive analytics focus on what could happen next based on past and present data, so that cities and regions can be best prepared to take appropriate action, while prescriptive analytics deal with the latter, i.e., with identifying the best course of action to take in response to an anticipated issue from among one or more available alternative options and in light of any constraints, resource availability and other factors or requirements that should be considered [18, 19]. However, in applying analytics, one (or the software algorithms used) should not go beyond the actual statistical strength of the (big) data, and should accommodate sound ‘error bars’ around all inferred predictions [20]. Also, big data methods can only be truly useful when paired with more conventional forms of information collection, or what some researchers call ‘small data’ [13]. Cities and regions should avoid being lost in a costly flood of big data, by starting with (the right) questions, not with data, and by using the right-sized data and analytics (‘big data diet’ and ‘light analytics’) to answer those questions.

### Standards

IoT relies on a growing number of sub-technologies and subsystems that need to be seamlessly interconnected and interfaced with one another in real time. This can only be achieved through the adoption of adequate standards and protocols for measurement, communication, integration, interoperability and control [6, 13]. Logvinov describes five key components/requirements and the corresponding standards governing them, without which IoT and its connected ‘things’ would not exist: the need for smarter power consumption, storage and management; the need for stronger safeguards for privacy and security; high-performance microcontroller units (MCUs); sensors and actuators; and the ability to communicate [21]. Web standards are also essential to ensure that IoT open data repositories, applications and services are able to interact seamlessly wherever and whenever needed (‘data plumbing’ standards, to ensure big data flow smoothly, securely and reliably through all ‘data pipes’).

### Privacy and security

IoT data and device privacy and security are among the most pressing challenges facing IoT-driven smart cities [6]. Users of connected devices and appliances such as smart TVs are often signing up to privacy and end user agreements that, besides being too long to read and understand, offer no alternative choice: if the user declines the agreements, s/he cannot use the equipment [22]. Furthermore, connected cars and body-worn or implanted medical devices can be remotely hacked, putting human lives at risk, and calling for a more security-aware approach to designing future devices and services [23, 24]. Possible solutions to IoT privacy concerns include offering individuals an option to ‘opt out’ of syncing their data with third-party or public cloud databases and services and become their own service providers [25]. The European Union recommends that IoT networks give individuals the rights to their own data and that privacy-friendly default settings be developed on IoT products and services to give users more control over what information is shared with others [26]. Rogers proposes a ‘right to introspection’, that users should be able to know exactly what and when data are leaving a device and their own home. He also recommends that manufacturers implement micro-client-side policies on IoT devices that users can control (e.g., to set bounding values such as temperature limits, so that deliberate attacks aimed at sending equipment out of limits can be prevented), as well as hardware switches to allow users to physically turn off features such as network and location functions. The latter (hardware switches) would afford a visible indication of the physical state of a connected device (with no need for a software user interface), besides enforcing a local, hardware-controlled form of security that cannot be easily overridden remotely [22].

## Smart, healthy and happy cities: it is all about people, open data, digital inclusion and citizen-centric services

Besides linking a physical world of atoms to a mirror world of bits, IoT can link atoms (people) to other atoms (people) via bits, resulting in the formation of ‘smart(er) communities’ that are socially connected in new ways and potentially happier. Cities, but also less urbanised regions and the countryside, could all benefit from, and harness the power of, IoT to actively engage citizens in a smarter participatory governance of their region [27], empower them to better care for one another, and promote stronger social inclusion and community cohesion.

The term ‘Internet of Things’ is sometimes seen as dehumanising, as it classifies ‘people’ under the same ‘bucket’ with other ‘connected things’, making no explicit distinction between the two [28]. People are not mere ‘connected objects’. People should be IoT’s *raison d’être* and its real ‘head’. Perhaps a better term here would be the ‘Internet of Things *and People*’. IoT-driven smart cities are most successful and smartest when their focus is on people, and when they actively involve and engage their citizens in co-creating (co-designing, co-developing and co-producing), co-running and co-monitoring (e.g., ‘citizen sensing’ [13, 29, 30]) the very smart services that are meant for them and for improving their living environment and overall quality of life. Indeed, a smart city is a smart community of people; opening up public data to citizens is one of the main keys to achieving this [14, 30].

Digital inclusion (e-inclusion) programmes such as ‘training the trainers/digital community ambassadors’ and Internet training programmes targeting older and disadvantaged people [31] thus become a necessity to mitigate a digital divide in the community, so that even the most technologically challenged among us is included and involved in the latest digital developments. e-Training older people can also be key to refranchising this important segment of the population, helping them contribute positively once again to the economy and live a more active and enjoyable life by re-entering the workforce in new, less demanding ways if they so wish (cf*.* e-labour exchange on Elance [32], doing some paid professional translation work or running some other Internet-mediated service at home). The European Digital Single Market [33] would further enhance the possibilities and opportunities in this respect for all age groups, and not just older people.

### Linking people to people – Vincles BCN and other examples

**Figure 1:**
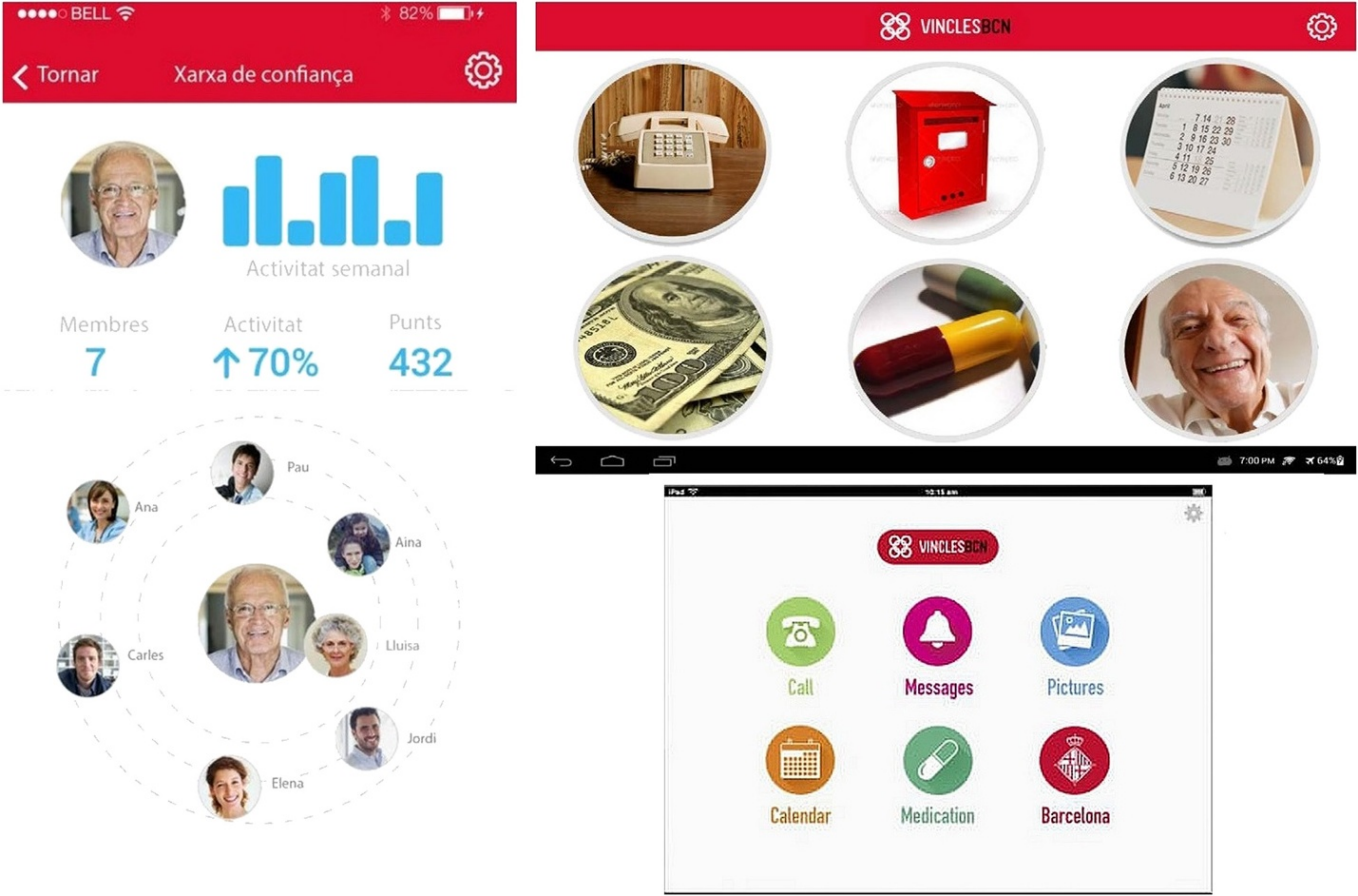
**Vincles BCN mobile app user interface for older people (** ***Ajuntament de Barcelona*** **).**

Helping and supporting others can make people happier. Lee describes how IoT can help users form a variety of communities of practice (smart communities) for enhancing the quality of life and curating available expertise in the community, giving the example of a smart home community-based service to allow collective baby and child care among neighbours [34]. At the Internet of Things World Forum held in Barcelona in October 2013, CISCO demoed a ‘car sharing service’ that combines social interactions (people offering to help each other, driver ratings, incentives, etc.) and IoT technology (location services, ride advertising and matching services, etc.) under one platform [35]. More recently, in September 2014, an Internet project by the Spanish city of Barcelona to reduce the social isolation of older people won the first European edition of Mayors Challenge, a competition designed to recognise novel ideas for solving the main challenges facing cities such as population ageing [36]. The competition is organised by Bloomberg Philanthropies, a foundation presided over by Michael Bloomberg, former mayor of New York. ‘Vincles BCN’, the winning project, is a mobile app aimed at helping Barcelona’s older people develop and maintain stronger social ties and networks with trusted and secure circles of social workers, volunteers, neighbours, friends and family. The app allows older users to engage in activities with their trusted networks, such as making calls, sending and receiving multimedia content, sharing a calendar and transferring money easily and safely, so that they no longer feel isolated or lonely, and can more easily find help when they need it, while at the same time continuing to live independently (Figure 1) [36, 37]. Research evidence shows that Internet use and social engagement can also protect against health literacy decline during ageing, independent of cognitive decline [38].

## Ensuring no one is left behind: smart countryside and the concept of ‘distributed cities’

While acknowledging that there is ‘no-one-size-fits-all’ IoT-driven smart city solution for cities of different sizes, needs and settings, the ‘economies of scale’, determined by the size of the population to be served by a given IoT service, usually play an important and decisive role when deploying sustainable smart services that are cost-efficient and cost-effective, and thus good value for taxpayers’ money. Smaller towns and villages tend to be left at a disadvantage because of their much smaller population sizes and modest local capacities and resources, all of which make the deployment of some IoT-driven smart services prohibitively expensive and non-viable at such limited scales. However, there is no reason why the populations of smaller and rural settlements should be left lagging behind, especially when one considers the negative consequences in terms of the difficulty these regions have in retaining their best local skills and stopping the eventual ‘brain drain’ of young professionals to the surrounding larger and more attractive cities.

### The ‘distributed city’ model

It has been said there is ‘power in unity’, and this cannot be truer than in the case of small neighbouring towns and villages (e.g., the Scottish Highlands and Islands, as well as sparsely populated regions in Nordic countries such as Finland) uniting together to reach the required economies of scale when their local populations and resources are pooled together under some suitable single administrative umbrella to form a larger ‘distributed city’. But for this pooling and the subsequent deployment of smart services to occur and succeed, the smaller settlements within a distributed city will need to be well connected with each other and the rest of the country by a good network of physical roads and/or highways and most importantly by reliable, high-speed cable and mobile Internet (cf*.* a metropolis and its suburbs consisting of multiple smaller cities and towns).

A distributed city of this kind can potentially become a ‘magnet’ for young and skilled professionals, as the countryside and smaller towns are converted into more attractive and digitally connected settlements, with new economic growth opportunities and better services akin to those found in larger cities and metropolises, thus removing all the negative connotations associated with rurality, while preserving the countryside’s unique good character (e.g., the quiet and beautiful nature, fresh air and less crowded public spaces) that is still preferred by many.

## Measuring and benchmarking smart cities and their impact on well-being

In January 2014, Antoni Vives, Barcelona’s Deputy Mayor for Urban Habitat, reported that his city was able to make big savings of more than €75 million in areas such as smart water, lighting and parking management, besides creating 47,000 new jobs related to smart city developments [13]. A January 2014 report prepared for the European Parliament’s Committee on Industry, Research and Energy, and entitled ‘*Mapping Smart Cities in the EU*’ [14] analysed a sizeable sample of smart city projects and initiatives across 37 European cities, including larger cities such as Barcelona, Budapest, Hamburg, London, Milan, Munich and Vienna. The report identified three key factors for successful smart cities (vision, people and process). These are: (i) the presence of a vision of inclusion and participation to avoid polarisation between urban elite and low income areas; (ii) the presence of inspiring leaders or ‘city champions’ who are able to foster participative environments, bringing together businesses, the public sector and citizens, with a focus on empowering citizens through active participation to create a sense of ownership and commitment; and (iii) the presence of a sound process, including the creation of a central office acting as go-between diverse stakeholders, open data provision, and various level of coordination and integration mechanisms (central and local) across ideas, initiatives, projects and stakeholders. The report also features summary dashboards for 20 shortlisted smart cities from among the 37 cities that were considered. The dashboards, found in Annex 10 of [14], offer information about eight areas for each of those 20 cities: basic city data, position *vis-à-vis* Europe 2020 [39] targets, city profile and innovation strategy, ICT (Information and Communications Technology) resources in place (capacity to pursue smart city initiatives), initiatives associated with the city (the dashboards cover 88 initiatives spread across the 20 cities), impacts expected from those initiatives, manifested smart city characteristics, and alignment to Europe 2020 targets.

### European Smart City Model Version 3.0 (2014)

**Figure 2:**
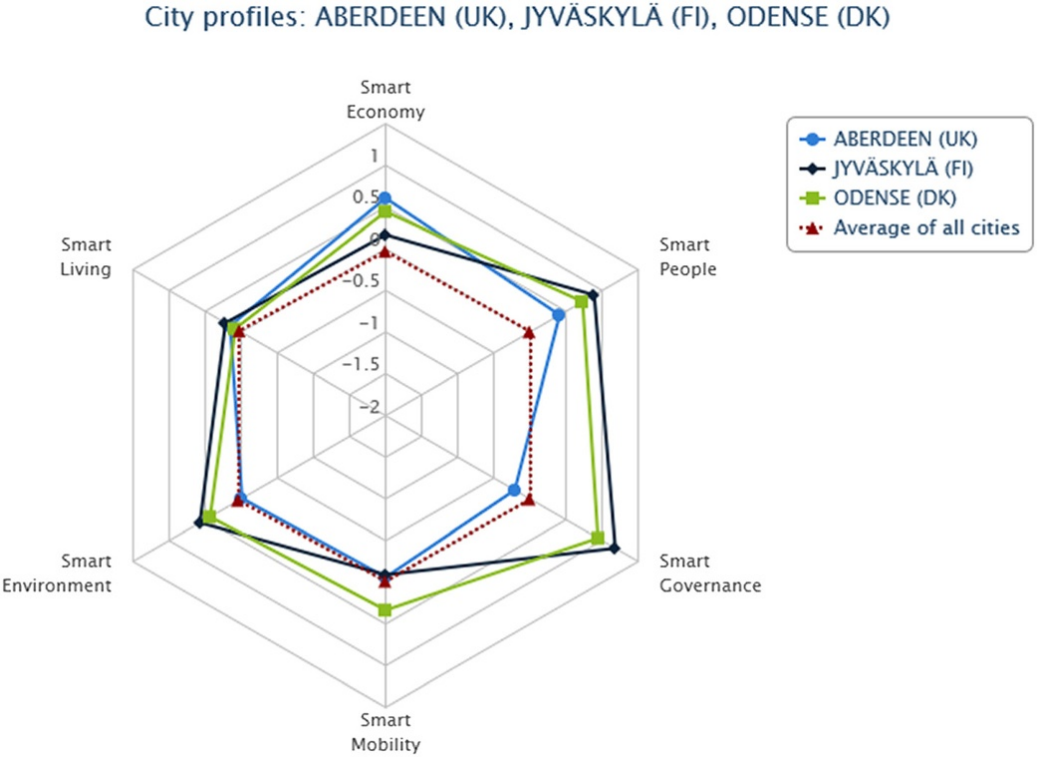
**The cities of Aberdeen (UK), Jyväskylä (FI) and Odense (DK) compared using the European Smart City Model for medium-sized cities Version 3.0, 2014 (TUWIEN/PLEEC).**

The European Smart City Model is a performance ranking and benchmarking tool for medium-sized cities with an urban population between 100,000 and 500,000. Version 3.0 (2014) of the tool covers 77 European medium-sized cities; larger cities and metropolises, such as the Spanish city of Barcelona, are excluded from the model by design. The model considers a city to be smart if it is performing well in six areas, namely Smart Economy, Smart Mobility, Smart Environment, Smart Governance, Smart Living and Smart People. Cities can be ranked and compared according to how well each city is performing in these areas (Figure 2). The tool was developed by the Department of Spatial Planning/Centre of Regional Science (*Fachbereich Stadt- und Regionalforschung - SRF*) at Vienna University of Technology (*Technische Universität Wien*) within PLEEC (PLanning for Energy Efficient Cities), an EU-funded Framework Programme 7 (FP7) project [15].

### OECD Better Life Index and Regional Well-Being

**Figure 3:**
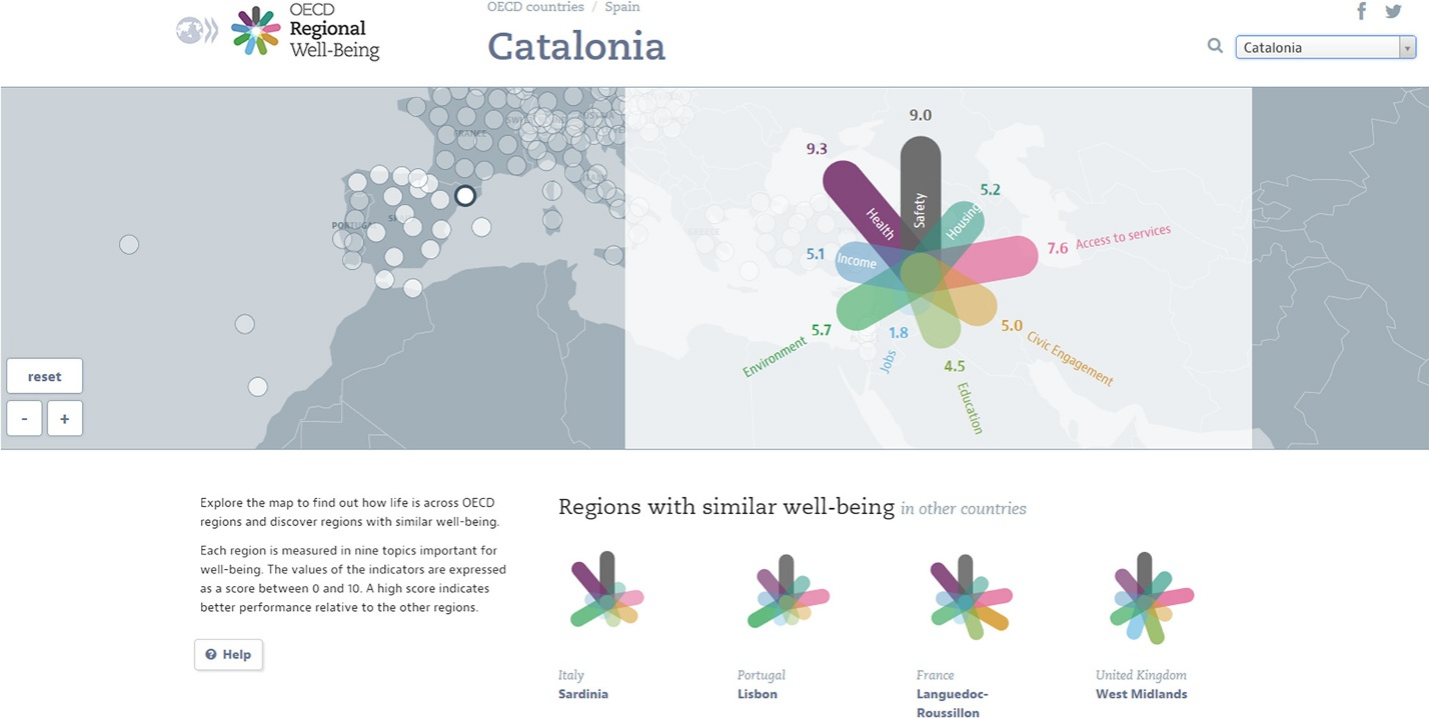
**OECD Regional Well-Being example: Catalonia, Spain.**

The ultimate outcomes of happiness and well-being can be more tricky to measure, but the OECD experience in this respect is quite notable, with their Better Life Index that goes beyond the cold numbers of GDP (Gross Domestic Product) and conventional economic statistics, covering 11 areas of material living conditions and quality of life, namely Housing, Income, Jobs, Community, Education, Environment, Civic Engagement, Health, Life Satisfaction, Safety and Work-Life Balance. Each of these topics affects people’s well-being and happiness in varying ways, and is measured by one to four indicators. Some, but not all, of these topic indicators are based on self-reported and other subjective data, e.g., from surveys that measure life satisfaction and happiness. The indicators attempt to capture those aspects of a topic that are most relevant to producing well-being under that topic category [8, 40]. For example, Education (one of the 11 topics covered) may help individuals live longer, participate more actively in civic life, commit fewer crimes, rely less on social assistance, and be less affected by unemployment trends. But the *quality* of education is what really matters the most when considering the positive effects that education can have on people’s lives. Rather than just measuring graduation rates, which while important, tell very little as to the quality of education received, the Better Life Index measures Education as a function of both educational attainment and student skills (the latter are computed from the results of OECD’s Programme for International Student Assessment or PISA, and serve as a proxy to quality of education) [41]. One of OECD’s key aims in developing this index was to engage the public in thinking about what makes a better life. Weights from 0 (not important) to 5 (very important) can be assigned to topics. The online application that builds the Index uses a default weight of 1 for each of the 11 topics, but users can build and customise their own Index by assigning their own weight values to each topic, based on how they rate each topic from 0 to 5 [8, 40]. The Better Life Index is, however, limited to life in the 34 OECD member countries. The related OECD Regional Well-Being--‘How’s life where you are?’ tool covers nine well-being dimensions (Access to Services, Civic Engagement, Education, Jobs, Environment, Income, Health, Safety, Housing) in 362 OECD regions rather than individual cities. OECD defines regions as the first tier of sub-national government, e.g., the region of Catalonia in Spain (Figure 3), rather than the city of Barcelona [42, 43].

## The Finnish experience in smart cities and related areas of innovation: brief highlights

The digital revolution can be harnessed as a great tool for tackling various inequalities, but this is not yet the case in many countries. The World Wide Web Foundation’s Web Index 2014–15 Report [44] evaluated the relations between the Web and inequalities throughout the world. The report’s Web Index ranking covered 86 countries, measuring the economic, social and political benefits that countries are gaining from the Web. In the 2014 ranking, the Nordic countries, Denmark, Finland and Norway, dominated the top three positions for the third year running.

The Global Liveability Ranking and Report (August 2014) by the Economist Intelligence Unit ranked Helsinki eighth in the Liveability Ranking (and second in Europe after the Austrian capital, Vienna, which ranked second in the Liveability Ranking) [45]. Helsinki has a number of exemplar smart city solutions and programmes in place, including HIR (Helsinki Region Infoshare), an open data platform that has now become part of the municipalities’ normal operations [14, 46]. But Helsinki is not the only smart city in Finland. The country’s Innovative Cities Programme (*INKA - INnovatiiviset KAupungit*, 2014–2020) run by Tekes, the Finnish Funding Agency for Innovation, aims at creating competitive, high-tech companies and top-notch talent to promote the emergence of innovation clusters in Finland capable of developing brand-new products and services for an international market. Five themes were selected for the programme: Smart City and Renewable Industry, Bioeconomy, Sustainable Energy Solutions, Future Health, and Cybersecurity. An urban region was appointed to take charge of each theme (e.g., Tampere for the Smart City theme and Oulu for Future Health), with seven other urban regions selected to partner in the five themes [7, 47, 48]. Finland has also placed an EIP-SCC initiative entitled ‘Northern Sparsely Populated Areas’ (NSPA). NSPA is an alliance of collaborating organisations and cities interested in clean energy in rural and remote areas of the EU [49].

An emerging field combining Finland’s long history of digitised healthcare information and the rapidly growing mainstream video gaming industry is ‘Games for Health’ [50–52]. These specialised games have desired health outcomes (e.g., [53]) and are used to encourage citizens to take responsibility for their own health and self-care. The ‘Games for Health Finland’ ecosystem (led by the city of Kuopio) integrates state‒of-the-art research, standardisation, safety, living labs (smart cities), user involvement and fast prototyping to promote entrepreneurship for global business [54]. This is a good example of how cross-sectoral collaboration around new technologies such as IoT and smart cities can create innovative solutions that motivate different user groups to achieve health benefits and at the same time reduce inequalities.

## Conclusions: Innovation and the fourth industrial revolution

Innovation is key to avoiding collapse and promoting sustainability of cities/regions and their infrastructures [13]. IoT for cities and regions is a major innovation, able to stimulate other forms of innovation, and in doing so, generate economic value, growth and competitive advantage. We believe that IoT will ultimately progress towards a ‘Plateau of Productivity’ after the current hype settles down [55], opening up many smart, viable and sustainable possibilities and opportunities to improve people’s lives in a diverse range of ways [56]. Quoting Matthew Jennings, Managing Director, Bosch Software Innovations, “*IoT represents the fourth industrial revolution, opening up opportunities for a more enjoyable life and new and better ways of doing business. While IoT will create entirely new business models, innovations will have to come more quickly for companies to stay relevant. To do this, managers need to envision the valuable new opportunities that become possible when the physical world is merged with the virtual world, and where potentially every physical object can be both intelligent and networked. Starting now, they must create the organisations and IoT-based business models that can turn these ideas into reality*” [57]. Cities and regions need to build their capacities in this respect. Postgraduate degrees in smart cities and urban informatics are increasingly offered these days by universities in developed countries to meet the demand for a ‘new workforce’ with new types of digital skills and expertise. The European Commission is leading a multi-stakeholder partnership, the Grand Coalition for Digital Jobs and Skills, to tackle the lack of digital skills and shortfall of talented ICT experts in Europe [58]. But ‘people first and foremost’ should be the ultimate goal of cities and regions, by enabling smart communities to develop and by empowering citizens, young and old, through active participation in this fourth industrial revolution of connected things and people. By focusing on people, smart cities and regions stand better chances of becoming healthier and happier cities, but it should be always remembered that technology is not a panacea but rather an enabler, and that other factors are equally important in creating happier and healthier cities and regions.

The 2015 Annual Healthy Cities Business and Technical Conference will take place in Kuopio, Finland, from 24–26 June 2015 [59], and it is hoped that the insightful discussions that started in Athens in October 2014 will continue and evolve further next summer in Kuopio.

## Electronic supplementary material

Additional file 1: **Slides: Creating self-aware and smart healthy cities – by MN Kamel Boulos.** (PDF 4 MB)
