# Supplementary material for: ‘Social, innovative and smart cities are happy and resilient’: insights from the WHO EURO 2014 International Healthy Cities Conference
Source: Int J Health Geogr. 2015 Jan 14;14:3. doi: 10.1186/1476-072X-14-3 (PMC4417221; doi:10.1186/1476-072X-14-3)
Supplement: Supplementary file 1 — Additional file 1: Slides: Creating self-aware and smart healthy cities – by MN Kamel Boulos. (PDF 4 MB) [file 12942_2014_635_MOESM1_ESM.pdf]

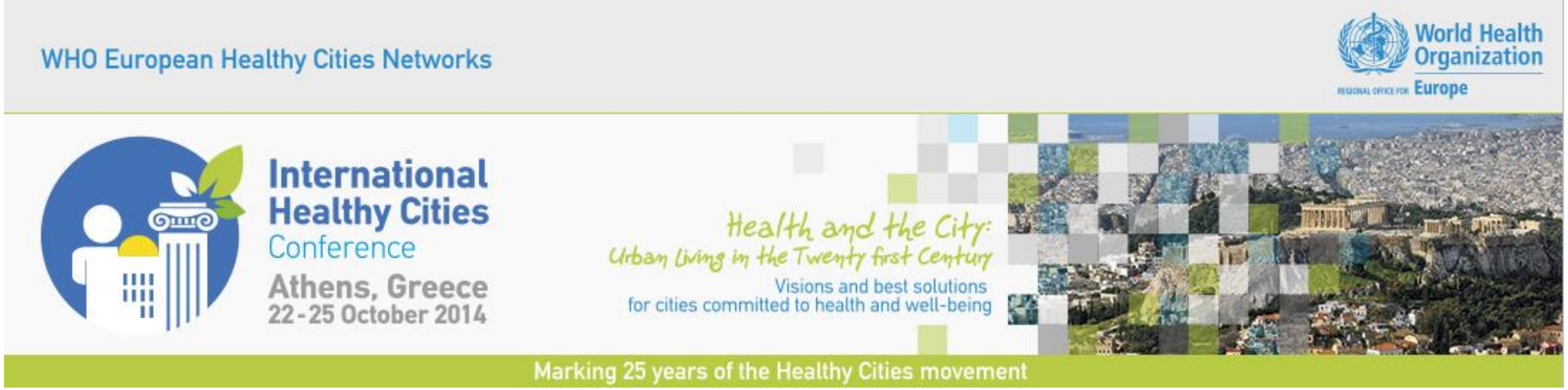

Creating self-aware and smart healthy cities

Maged N. Kamel Boulos, MBBCh, PhD, SMIEEE

Professor and Chair of Digital Health

The Alexander Graham Bell Centre for Digital Health

University of the Highlands and Islands, Scotland, UK

maged.kamelboulos@uhi.ac.uk
mnkboulos@ieee.org
twitter: @mnkboulos

University of the Highlands and Islands
Oilthigh na Gàidhealtachd agus nan Eilean

The Alexander Graham Bell Centre for digital health

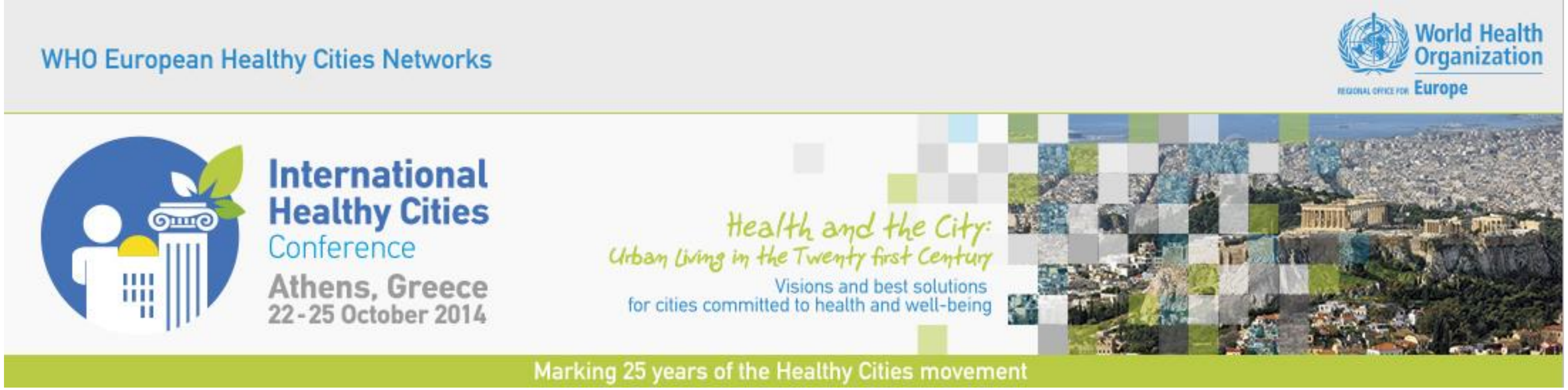

Agenda

- The Internet of Things (and People) (IoT): a primer with examples
  - IoT standards and protocols
  - IoT big data and analytics
- IoT-powered cities: towards self-aware, smart and potentially healthier cities
  - Case study: Barcelona and Sant Cugat (a suburb north of Barcelona)
- Concerns: IoT data and device privacy and security
- Conclusions

The Internet of Things (and People)

- The Universe, our bodies and the physical animate and inanimate objects around us have always generated enormous amounts of data, but we were not able to fully capture and make sense of those data until recently.
- Today, the Internet of Things (IoT) enables almost everything (people and objects on our planet and the Universe) to be automatically **identified and located**, e.g., using RFID or Radio-Frequency IDentification tags, **instrumented** using a very wide array of specialised **sensors** or detectors to capture data, and **connected / interconnected** to cloud servers for storage, processing and **'in context' / 'intelligent' analysis** of captured data.

'Internet of Things' was added to Oxford Dictionaries in 2013: http://www.oxforddictionaries.com/definition/english/Internet-of-things

Oxford Dictionaries
Language matters

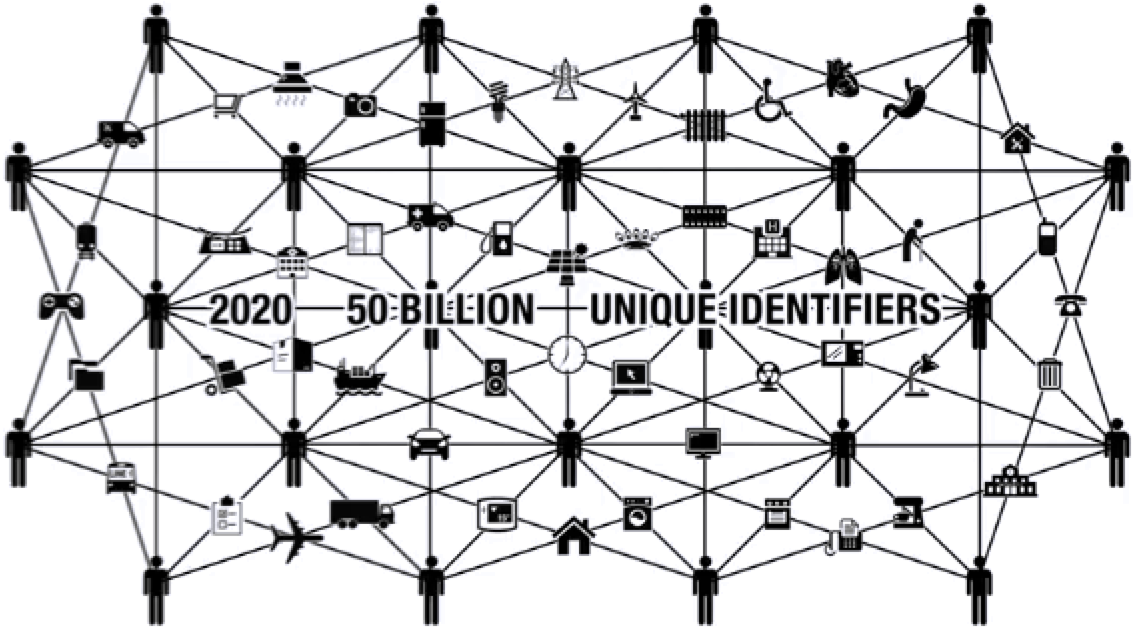

By 2020, 50 billion things and devices will be equipped with unique identifiers.

Image source: https://www.youtube.com/watch?v=qfhcPvwzJF0

The Internet of Things (and People)

- The result are **smarter 'things' and smarter environments** in which people and objects interact and cooperate with each other in better and new ways that can vastly improve our quality of life.
- A '**quantified self**' is now possible, thanks to a growing range of wireless sensors in gadgets / **wearables** (e.g., smart watches and smart garments) and implantables that can continuously monitor and track in real time various aspects of a person's daily life and clinical status.
- Internet-connected wireless health sensors and sensors around the home are key to modern **telehealthcare** services (also known as '**connected health/care**'), particularly those aimed at prolonging the **independent living** or 'ageing well' of older people (**Ambient Assisted Living—AAL**).

The 'smart garment' used in the **eCAALYX** EU-funded AAL project: http://ecaalyx.org/

- Biometric textile sensors integration
- Body temperature
- Heart rate
- Respiratory rate
- Wireless communication

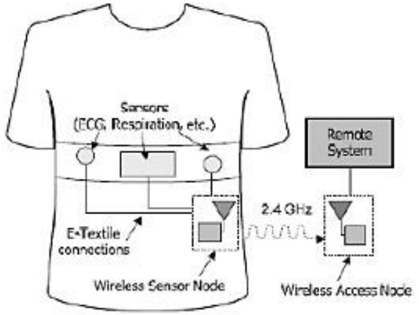

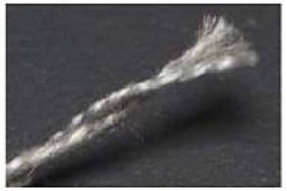

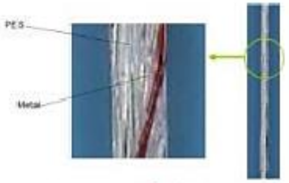

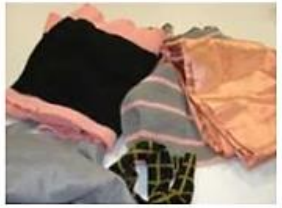

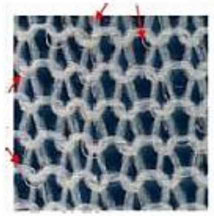

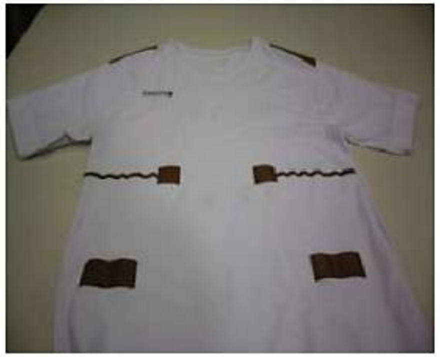

The Internet of Things (and People)

- Connected 'things' can also have **remotely-controlled actuators or other mechanisms** to trigger or modify **actions** carried out by those objects according to needs measured by sensors (or user-initiated), e.g., in home automation (domotics) scenarios.

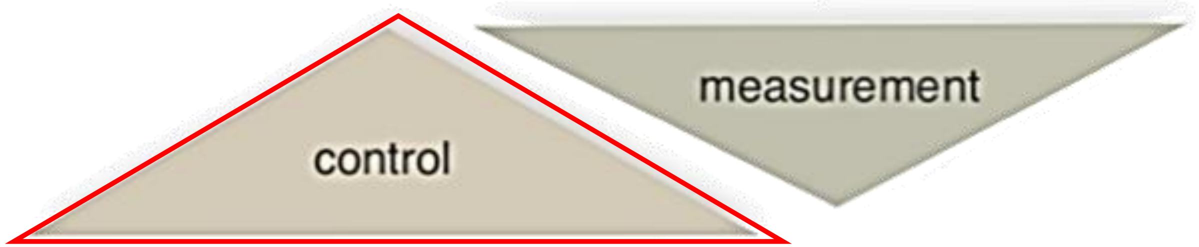

- [**Public engagement / citizen sensing / crowdsourced efforts**](): the 'Air Quality Egg' example - http://airqualityegg.com/.

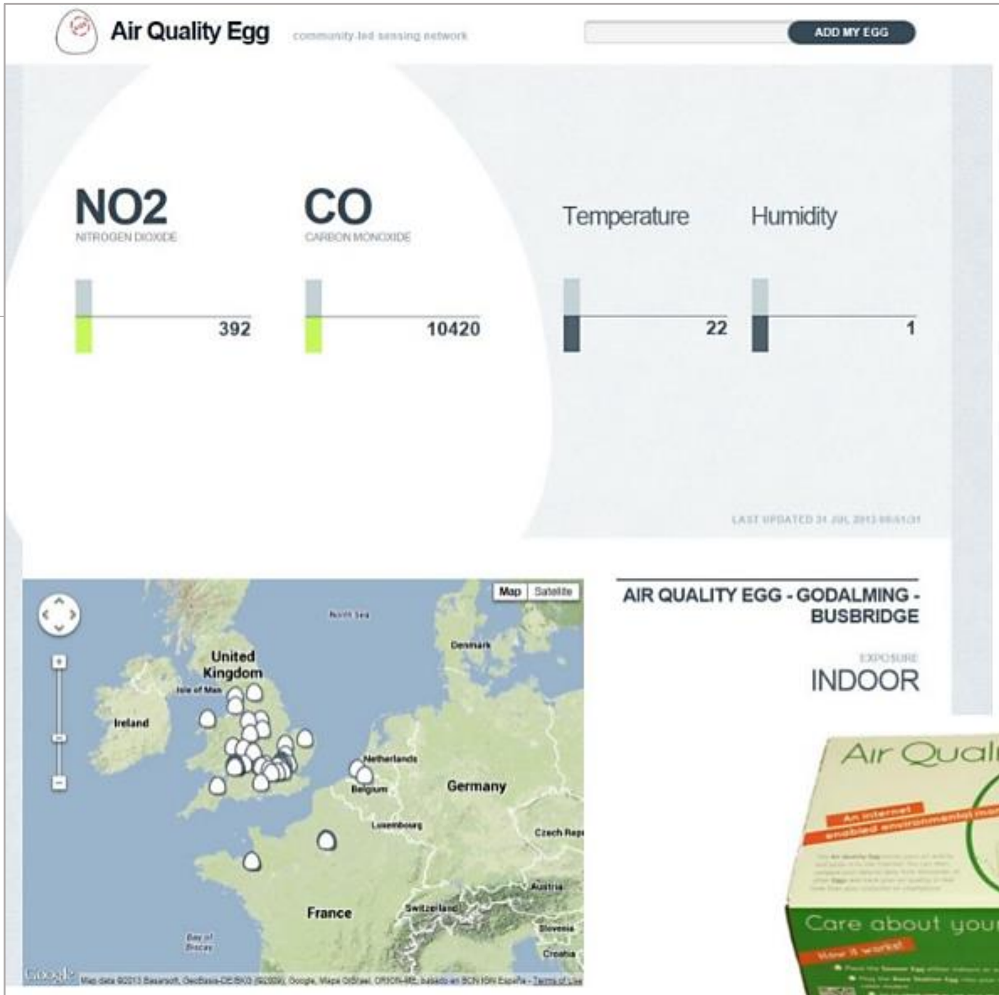

Community-led air quality monitoring around the globe, powered by **Xively**.

Anyone can buy an **Air Quality Egg** at http://wickeddevice.com/index.php?main_page=product_info&cPath=28&products_id=108 (US $185 per unit, as at 31 July 2013) and contribute to the global map.

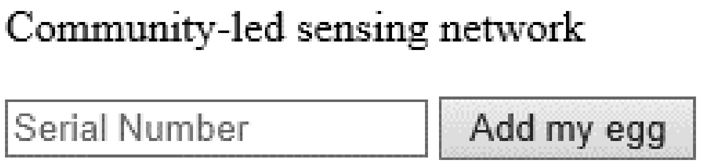

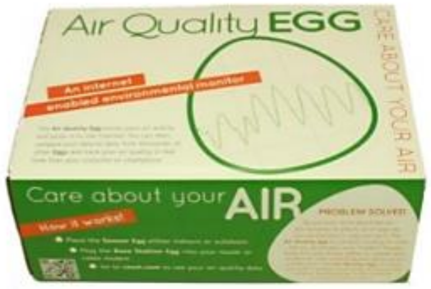

Air Quality Egg

Source (Kamel Boulos, 2013): http://www.slideshare.net/sl.medic/public-engagement-and-participation-in-health-geography

How to convert an object to a connected 'smart thing'

- Suppose we have a **chair**. We want to know (using an Internet-connected smartphone / tablet or computer) **from anywhere in the world if it is occupied, and who is currently sitting in it**.
- To convert the chair into a smart one, we first need to give it a **unique identity (IPv6)** to uniquely identify it and connect to it specifically (as opposed to all other / similar chairs in the same room or elsewhere).
- We then need to give it the **ability to communicate (wirelessly)** and equip it with the **ability to 'sense' its own status and its environment via sensors**.
- A **pressure sensor** on the seat can detect whether the chair is occupied or not. An **RFID tag reader** attached to the seat can identify any tagged person sitting in it.
- We can even think of **ways to control that chair, e.g., move it, from anywhere in the world**, using a **remotely-controlled actuator / motor** (though this does not make useful sense with the chair example).

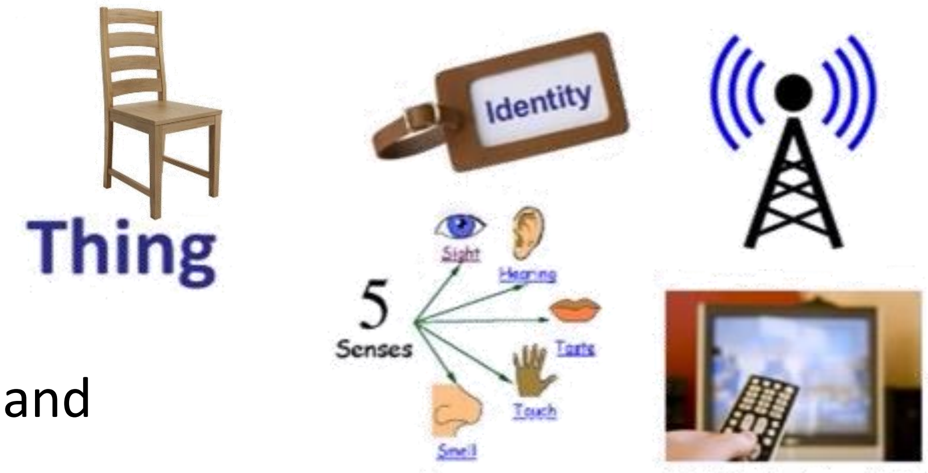

Image and example source:
https://www.youtube.com/watch?v=QaTIt1C5R-M
(**Dr John Barrett**, Cork Institute of Technology)

Let's play: an IoT simulator (by IBM)!

- Available at https://developer.ibm.com/iot/recipes/simulator/

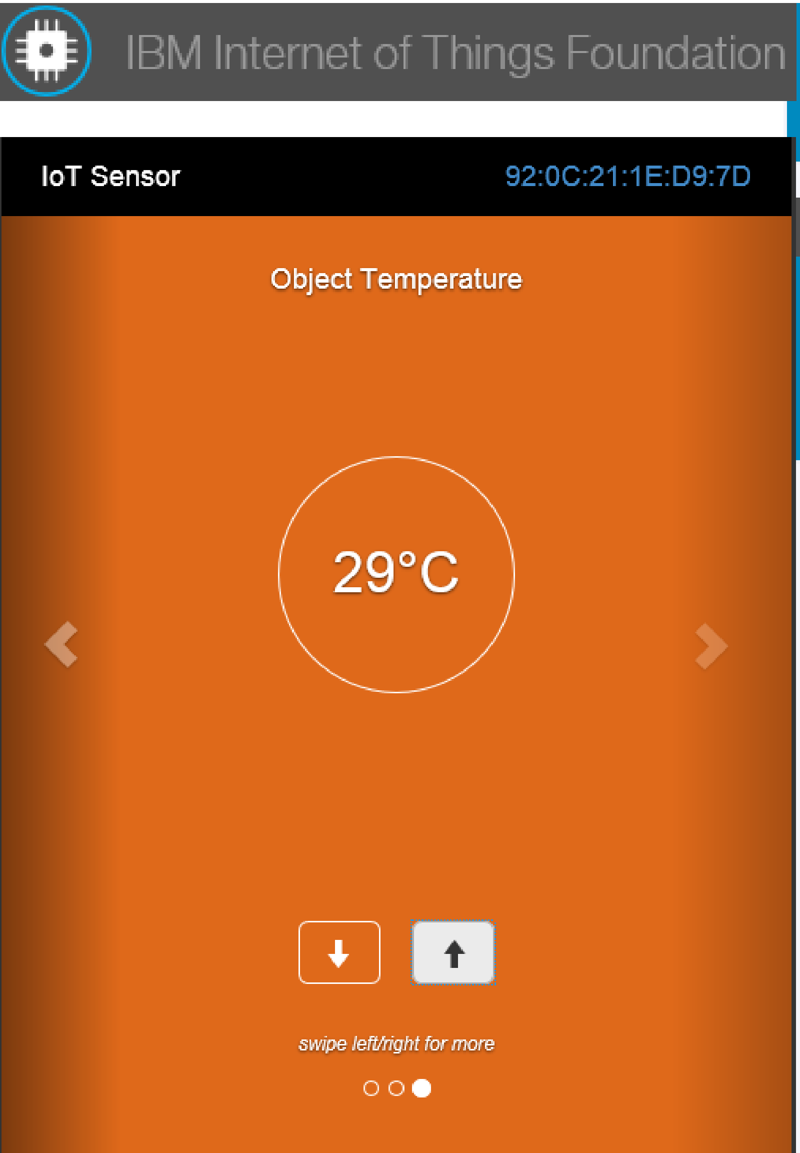

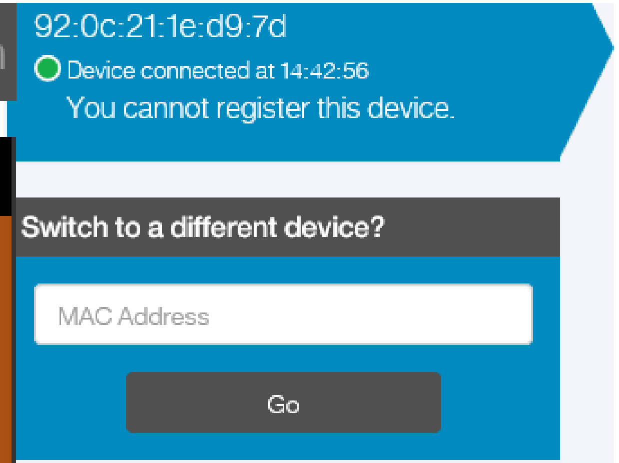

< Device (3 sensors) simulator and visualisation dashboard >

Device simulator and dash-board can run on two separate Internet-connected smartphones, tablets or computers (or on same machine).

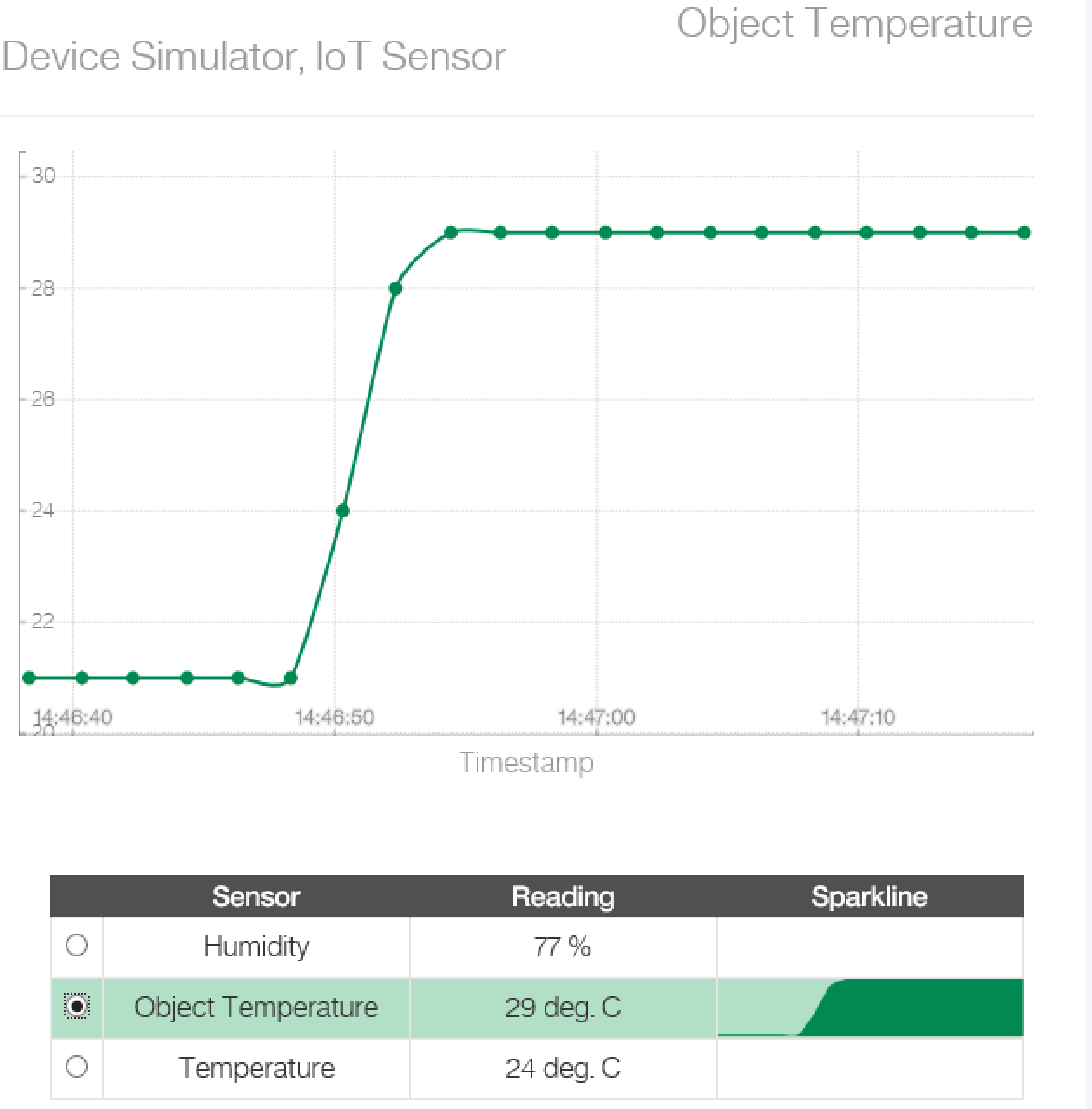

The Internet of Things (and People)

- **Boundaries between 'digital' (bits/data) and 'physical' (atoms/things) are getting blurred**:
  Internet-connected smartphones, phablets* and similar can also sense and 'recognise' where we are (**location awareness**) and the persons, places, things and objects around us (via camera/cloud-powered image recognition, QR [Quick Response] code scanning, etc.), and link them to relevant information (e.g., 'Layar' augmented reality app, ESRI 'My Place History' geomedicine app, Amazon Fire Phone and Google Glass), or even use them as elements of a digital game (or exergame) taking place in the real world!

* Phone-tablet

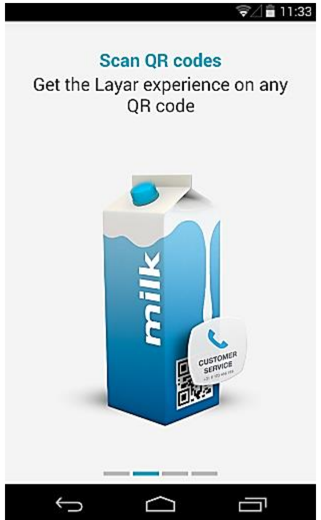

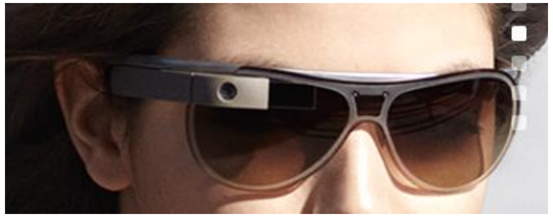

Video: IoT-powered (connected) pillbox

- Length: 0:38 - https://www.youtube.com/watch?v=RTdRUwl9JsA (also locally embedded on next slide)

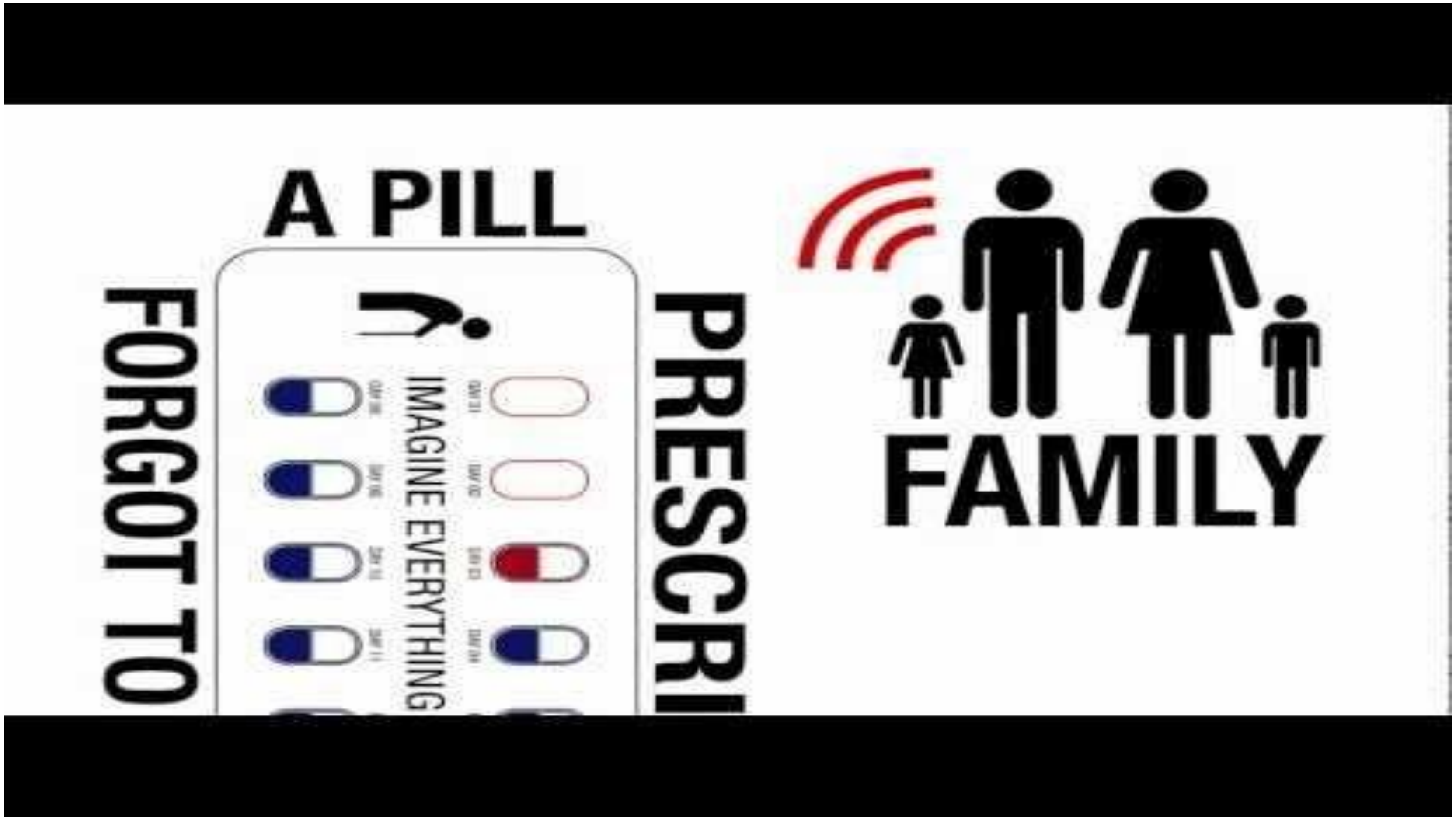

Embedded video is not available in this copy of the presentation. Please refer to the previous slide for the YouTube version.

Connected vehicles

- The emerging EU '**connected cars**' vision and standards (see http://europa.eu/rapid/press-release_IP-14-141_en.htm) have the potential of reducing traffic bottlenecks and road traffic accidents, in addition to improving ambulance response times.

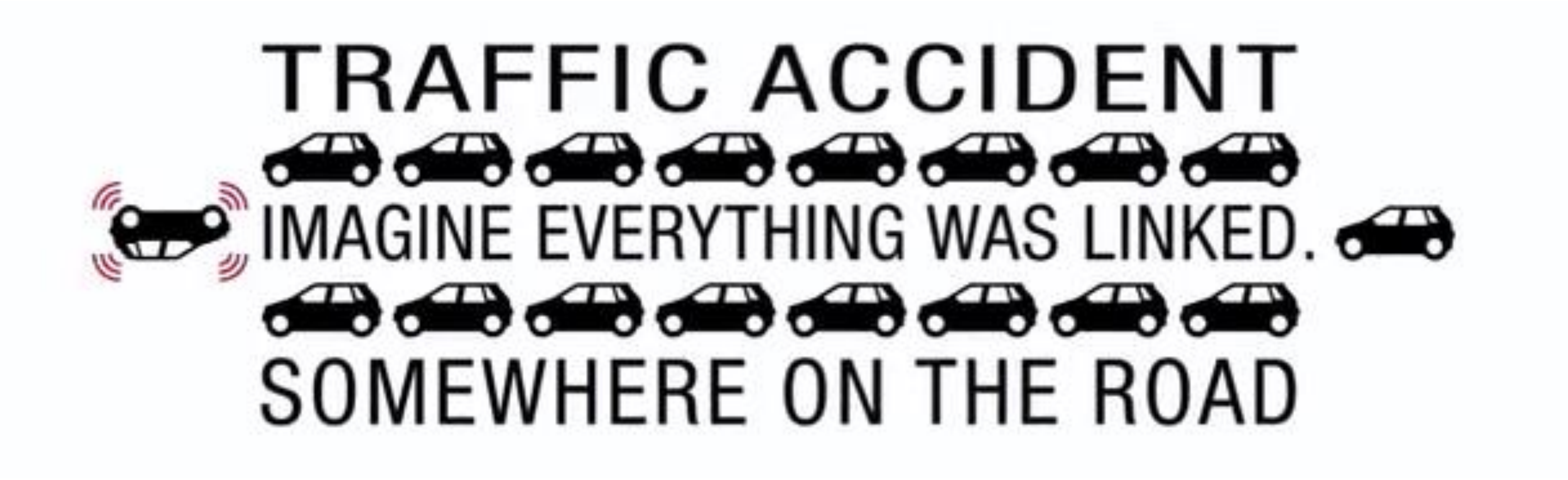

Image source: https://www.youtube.com/watch?v=lkIXHzcU5XA

Video: IoT-powered vehicles

- Length: 0:38 - https://www.youtube.com/watch?v=lkIXHzcU5XA (also locally embedded on next slide)

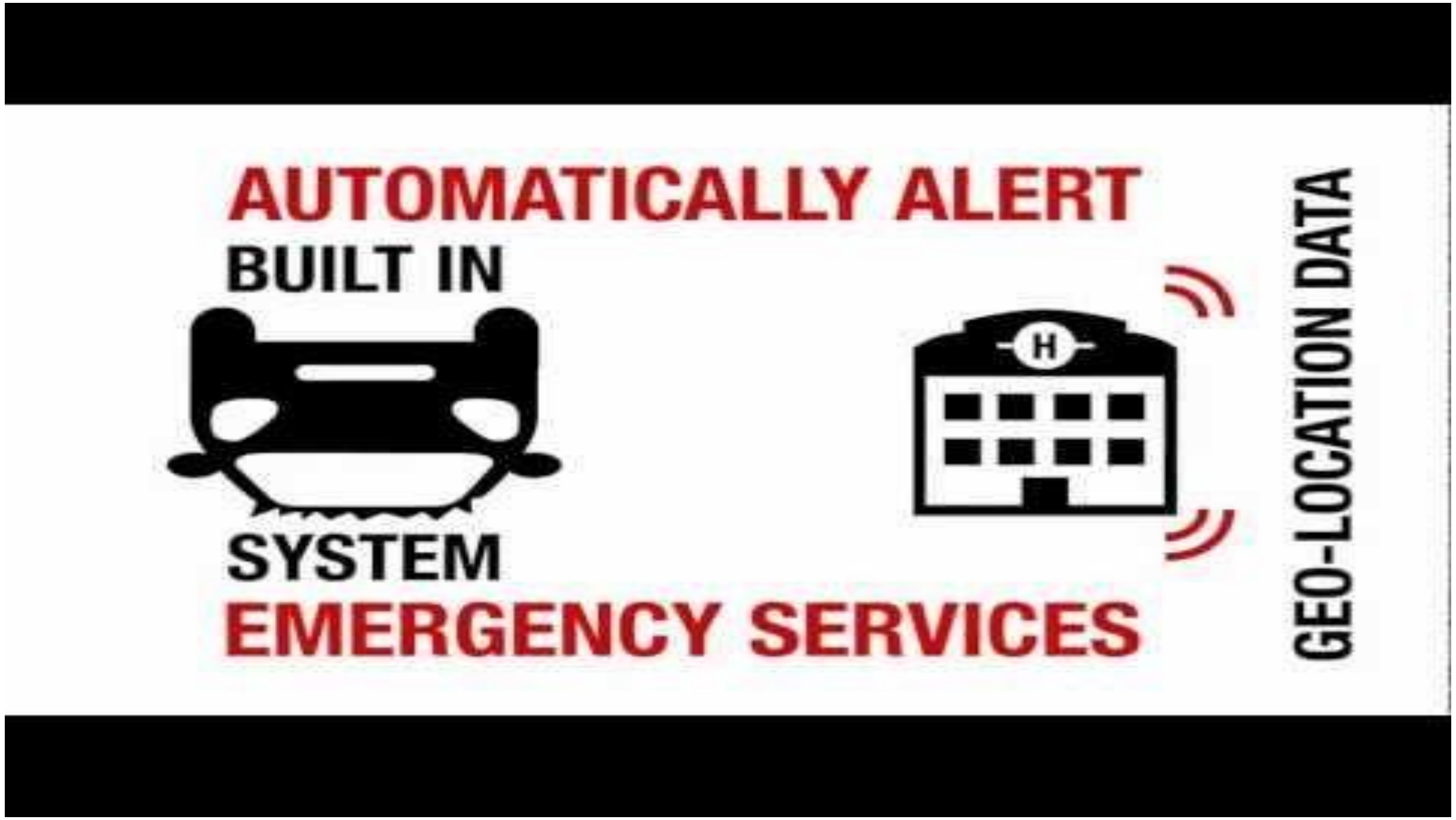

Embedded video is not available in this copy of the presentation. Please refer to the previous slide for the YouTube version.

Standards & protocols

- In IoT, everything has an IP address. **Internet Protocol version 6 (IPv6)** uses 128-bit addresses, allowing 2^{128}, or ~3.4×10^{38} addresses, i.e., >7.9×10^{28} times as many as IPv4, which uses 32-bit addresses and provides ~4.3 billion addresses.
- There are very many other protocols and standards required to make IoT work, e.g., IEEE 802.15.4 standard for low-rate wireless personal area networks (LR-WPANs) and ISO/IEC 18092 standard for Near Field Communication (NFC), [Continua Version 2014] (compilation of standards and specs for connected health / personal telehealth), among many others.

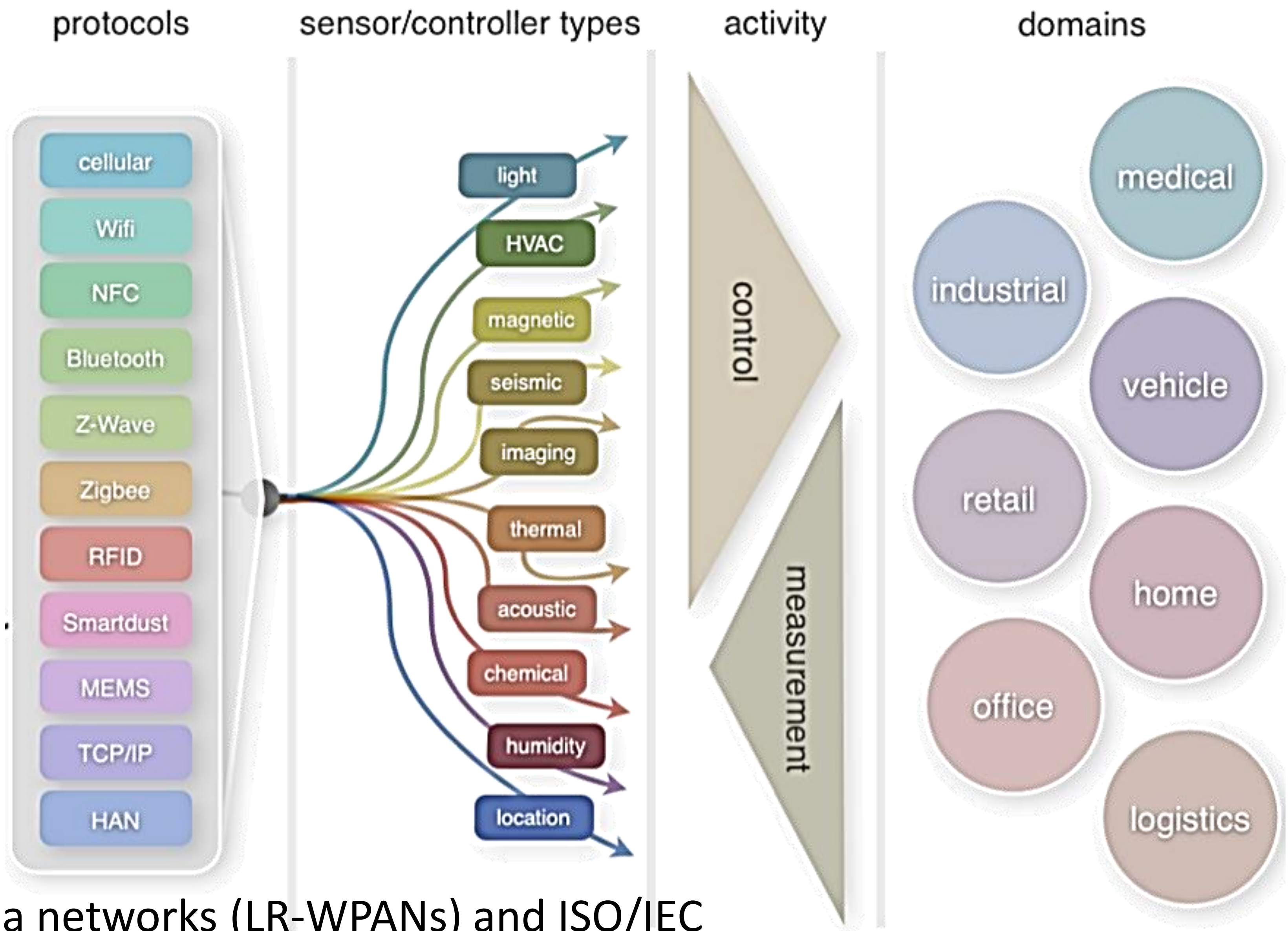

Image source: Adjuvi LLC
HAN: Home Area Network
HVAC: Heating, Ventilating and Air Conditioning
MEMS: MicroElectroMechanical Systems
RFID: Radio-Frequency Identification

IoT generates (and consumes) big data

- IoT-driven sensors, devices, systems and services generate big amounts of real-time data (not all of which are trustworthy and reliable).
- Much of the IoT-generated 'big data' are geo-tagged or **geo-located**. Location gives important context ('**where**'), as well as being an essential piece of information to know when responding to events and deploying appropriate actions (but also has some individual privacy implications).
- Big data and their context are key to gaining **new insights** that can **inform better decision making** in various scenarios. The importance of having robust, intelligent **analytics** systems in place to process and make sense of such data in real time cannot, thus, be overestimated.

Image sources: InformationWeek ('big data' elephant) and http://www.crowdsourcing.org/editorial/the-crowd-maps-beijing-floods-faster-better-than-chinese-government/17422 (Beijing floods, 7/2012)

The 4Vs of big data: According to Gartner (2012), "big data are high **Volume**, high **Velocity**, and/or high **Variety** information assets that require new forms of processing to enable enhanced decision making, insight discovery and process optimisation". A fourth V for '**Veracity**' can be added to the definition to emphasise the importance of establishing data trustworthiness and accuracy.

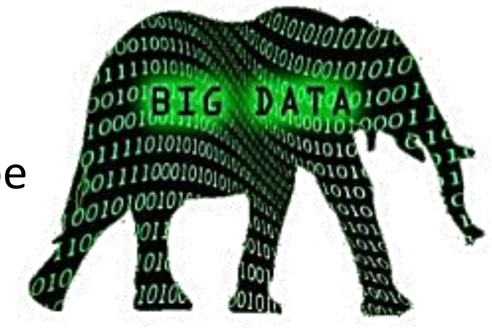

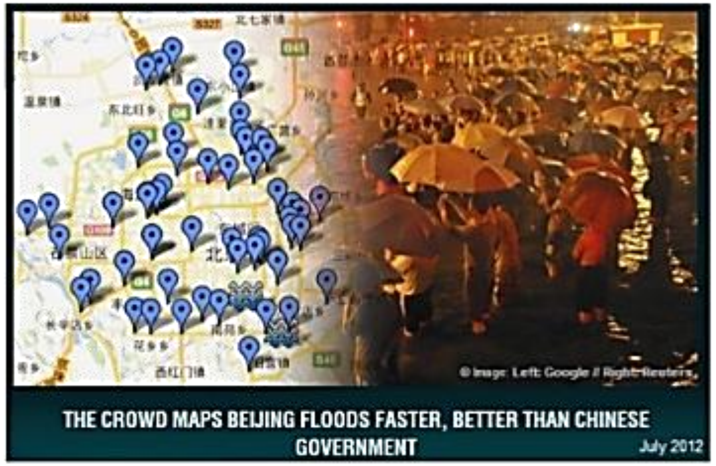

IoT big data examples

- Farmers can now use sensors to continuously monitor cows' health and track their movements, ensuring better milk supply for human consumption.
- On average, *each cow* generates 200MB of data/year!
- 'Quantified self' data from the growing number of personal clinical and health & fitness wearables / sensors available today can easily exceed the above figure (*per person*).

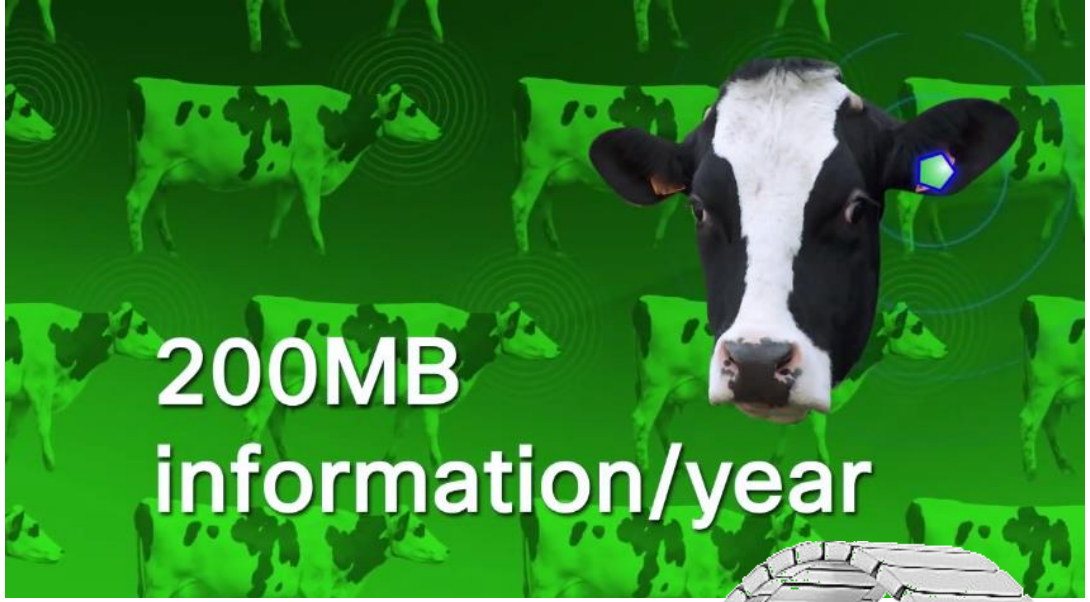

Image sources:
CISCO, Apple and Fitbit

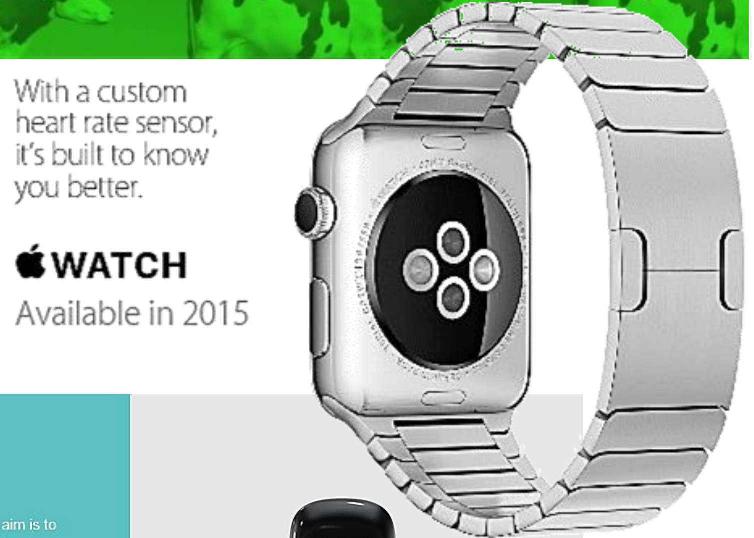

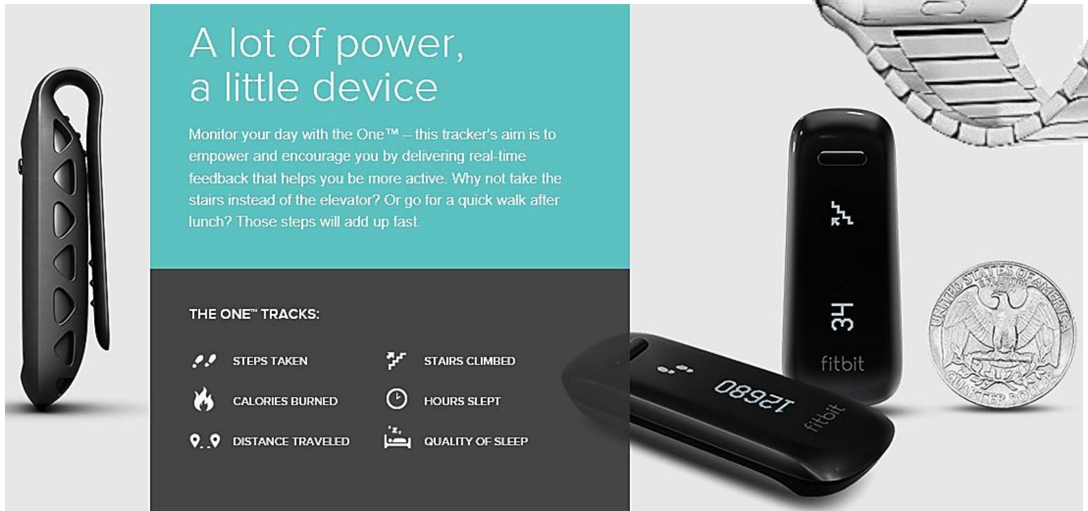

Big data analytics

- There are three main analytics levels:
  - **Descriptive analytics:** what has happened and where (smart location) / the 'big picture';
  - **Prescriptive analytics:** choices currently available; and
  - **Anticipatory / predictive analytics:** what could happen next and how to be prepared / take action.
- Health is geospatial, and if we can see trends spatially (**the 'where' / smart location component**), we can monitor and improve population and individuals' health.
- But do not go beyond the actual statistical strength of the (big) data; accommodate sound '**error bars**' around all inferred predictions.
- Also 'big data' methods can only be truly useful if they are paired with traditional forms of information collection, or what some researchers call '**small data**'.

Image source: IBM - https://www.youtube.com/watch?v=sfEbMV295Kk

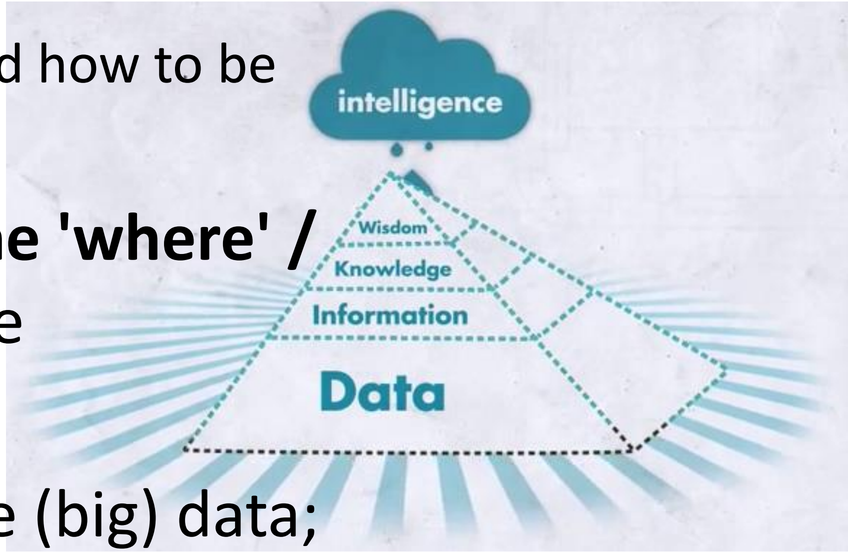

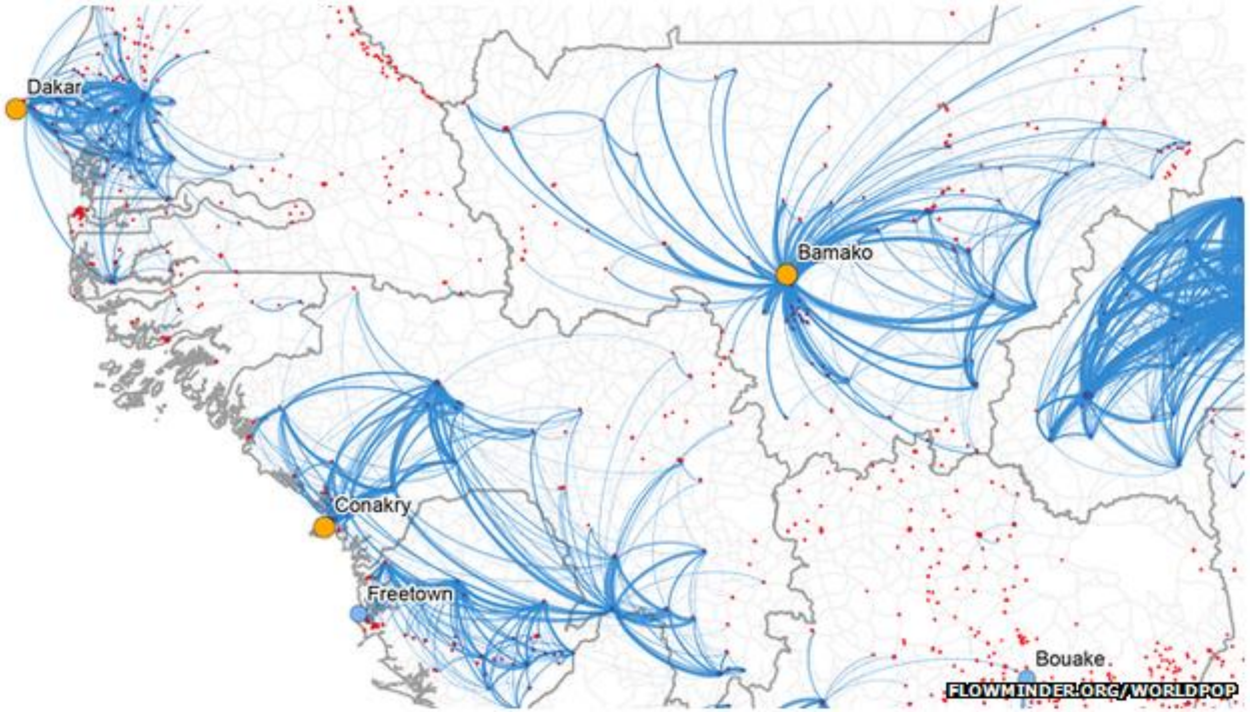

Mobile phone data from West Africa is being used to map population movements and predict how the Ebola virus might spread

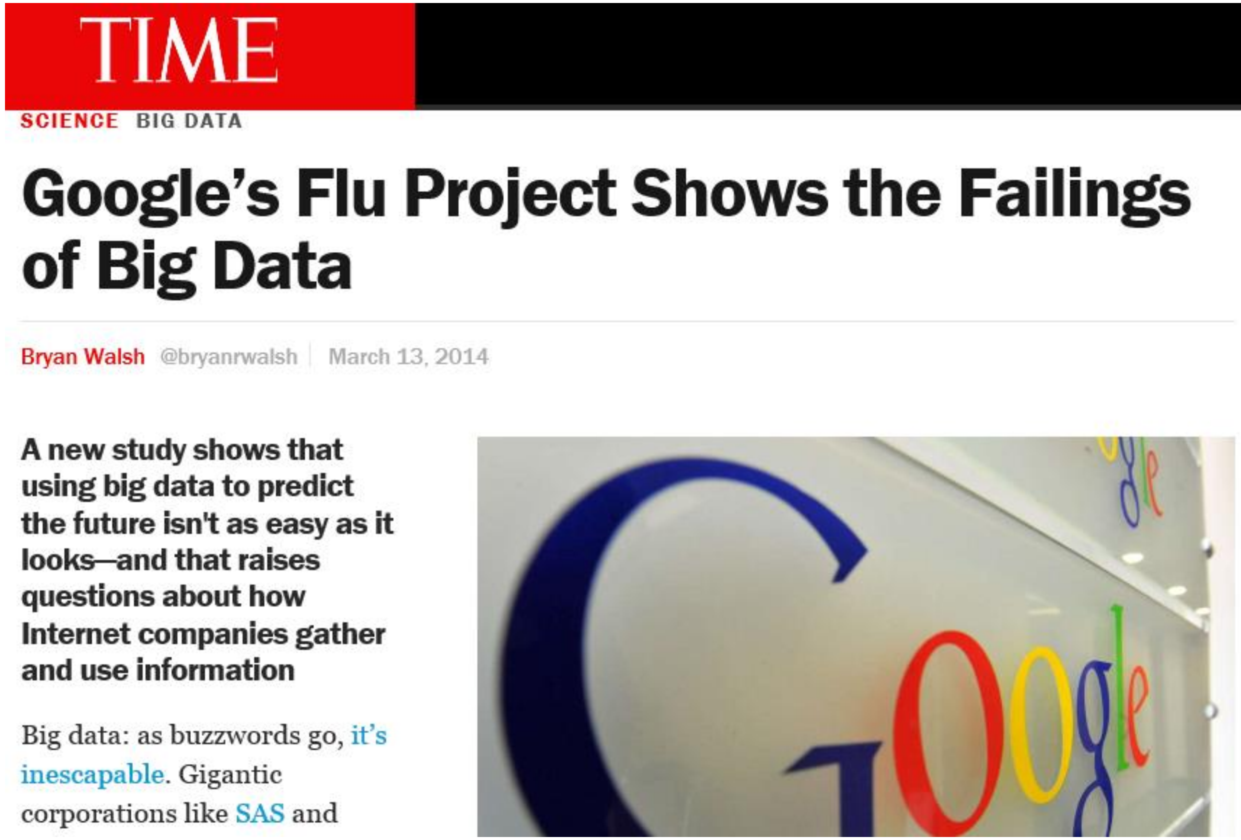
TIME

SCIENCE BIG DATA

Google's Flu Project Shows the Failings of Big Data

Bryan Walsh @bryanrwalsh | March 13, 2014

A new study shows that using big data to predict the future isn't as easy as it looks—and that raises questions about how Internet companies gather and use information

Big data: as buzzwords go, it's inescapable. Gigantic corporations like SAS and

- Wall M: **Ebola: Can big data analytics help contain its spread?** BBC News 2014 Oct 15, Online at http://www.bbc.co.uk/news/business-29617831
- Lazer D *et al.*: **The parable of Google Flu: traps in big data analysis.** Science 2014, **343**(6176):1203-1205.
- Gomes L: **Interview with IEEE Fellow Michael I. Jordan on 'Why (or when) big data could be a big fail'.** IEEE Spectrum 2014 Oct 20, Online at http://spectrum.ieee.org/robotics/artificial-intelligence/machinelearning-maestro-michael-jordan-on-the-delusions-of-big-data-and-other-huge-engineering-efforts#qaTopicThree

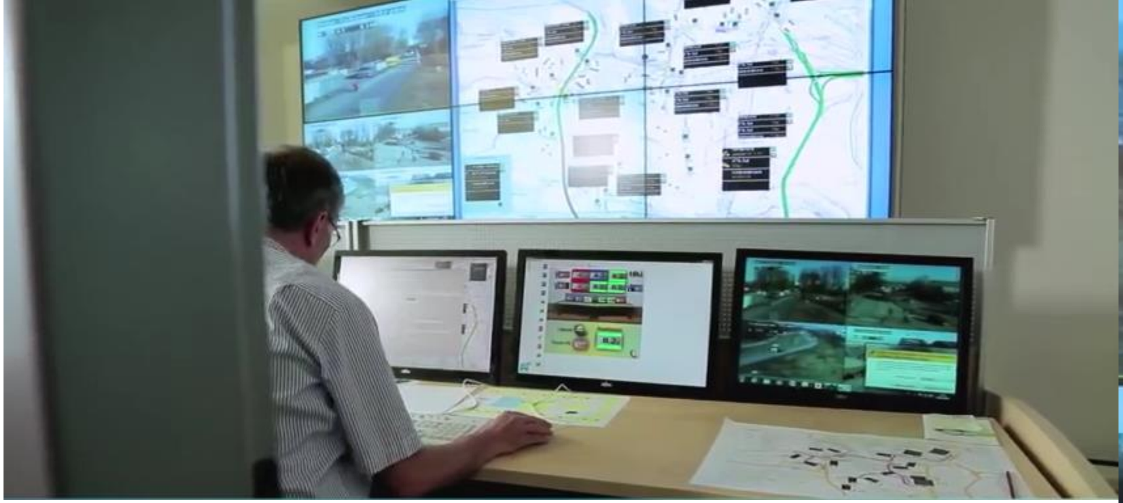

Dashboards and real-time visualisation

Smart traffic management in Hamburg, Germany

Image source: https://www.youtube.com/watch?v=FoEPlE8Pg7I

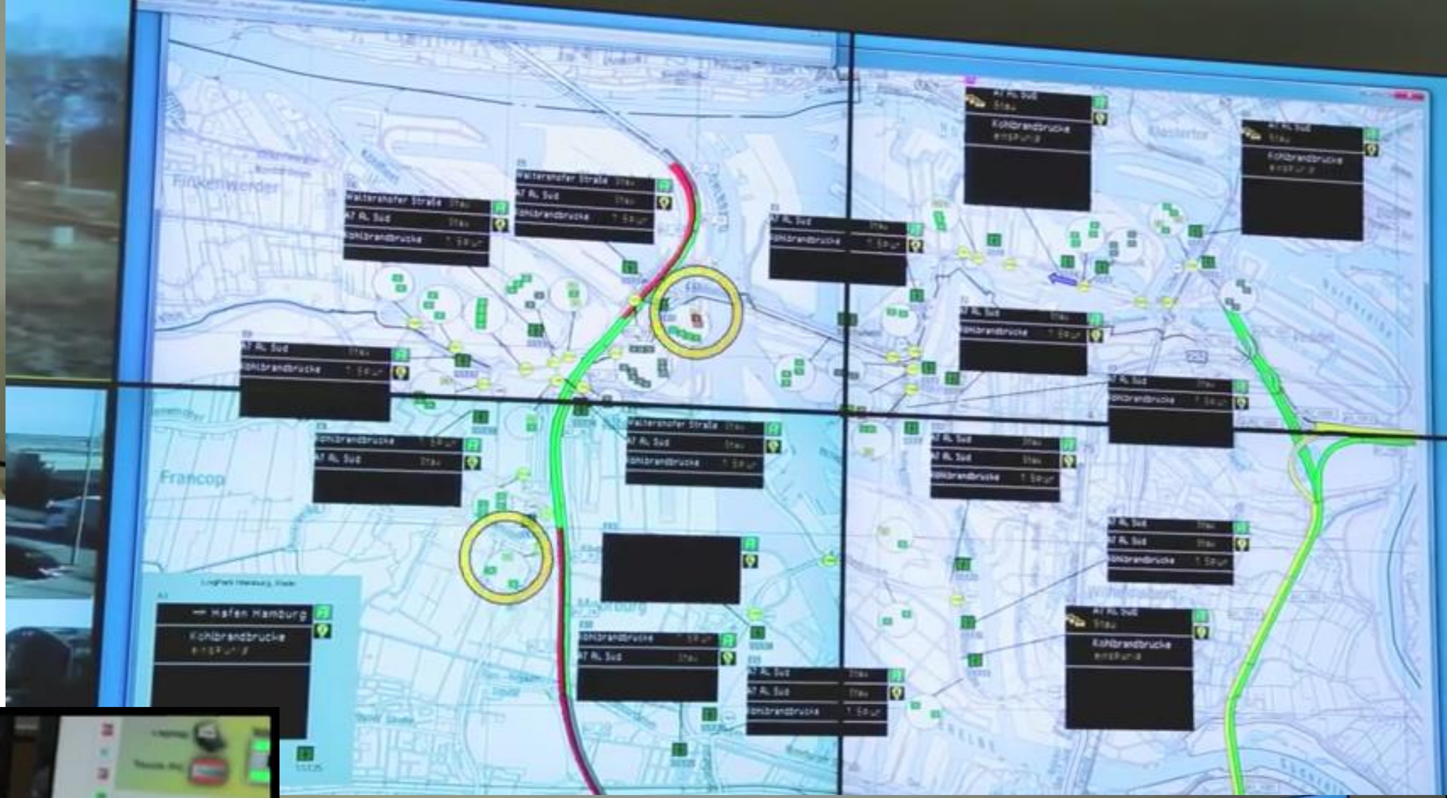

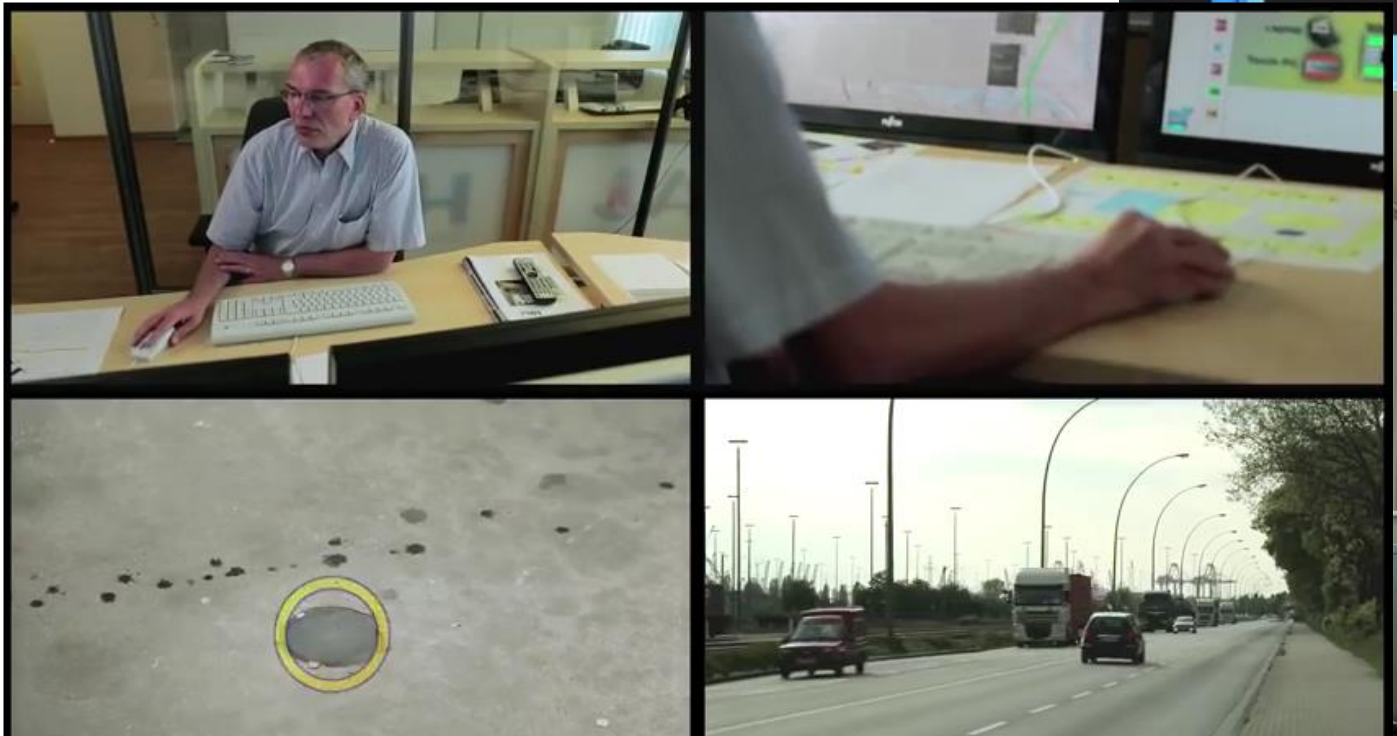

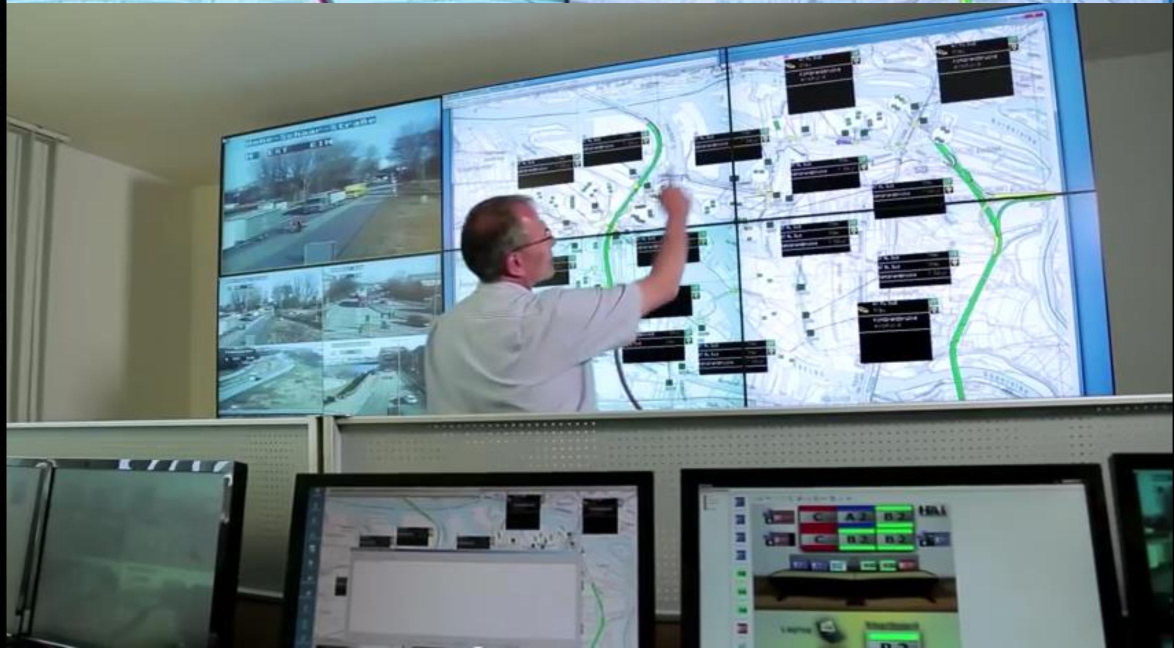

IoT-powered cities

- "*The cities of the future will be self-aware, much like a being. These cities will be able to reconfigure themselves, based on what's happening, and what might happen, in the immediate future.*
- "*You have two versions of cities. One is made of atoms. You and me, and cars, and all this sort of thing. We are atoms. Then you have another version which is a mirror city, which is made of bits. And the two are connected to one another. If you have sensors in the one, it can connect to the other.*"

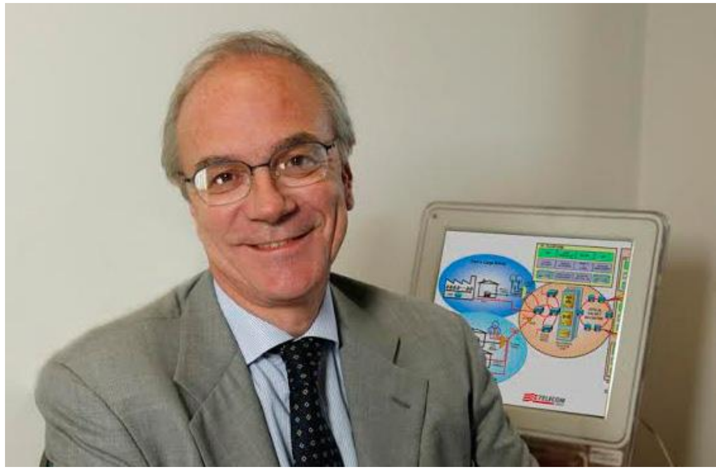

--**Roberto Saracco**, Chair of IEEE's (Institute of Electrical and Electronics Engineers) Future Directions Committee and EIT ICT Labs Italian Node Director

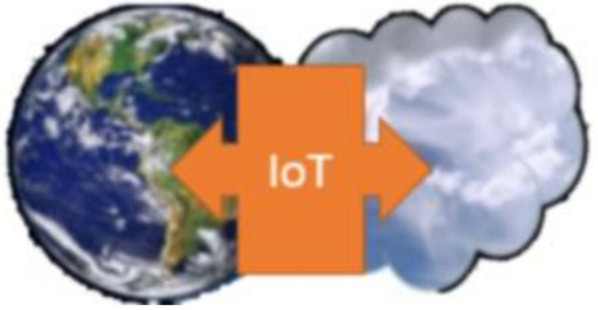

Barcelona: an IoT-powered smart city

- A 500-Km-long **underground fibre network** is being installed progressively as the city carries out routine maintenance to its roads and other underground services, which helps reduce installation costs significantly.
- Solar-panel-powered **smart bus stops** are connected to the city's fibre network. They display real-time bus information and times, tourist information and digital advertising, offer USB (Universal Serial Bus) charging sockets for mobile devices such as smartphones and tablets, and act as free WiFi (Wireless Fidelity) hotspots, allowing people to connect to the Internet using their mobile devices while waiting for a bus.

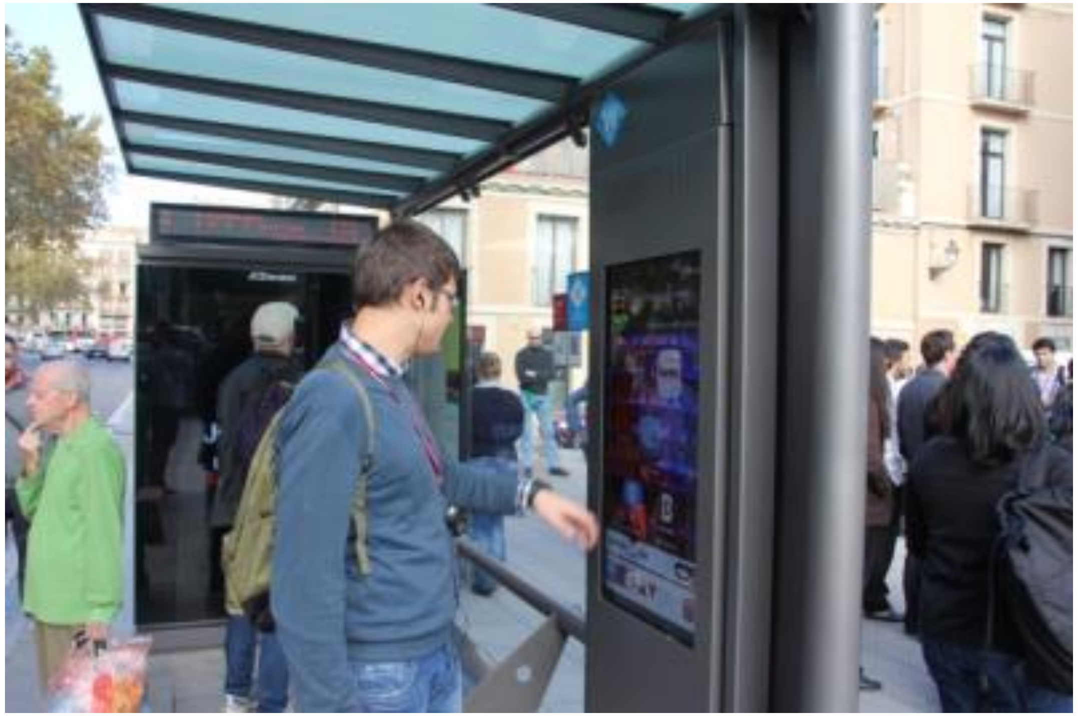

Image source: http://paulwallbank.com/2013/11/01/touring-barcelona-smart-city-internet-of-things/

Barcelona: an IoT-powered smart city

- The city's **smart parking spots** are also connected to Barcelona's WiFi network. They detect the presence of cars through a combination of light and metal detectors.
- Online searching and payment for the smart parking spots is possible using dedicated **smartphone apps**.

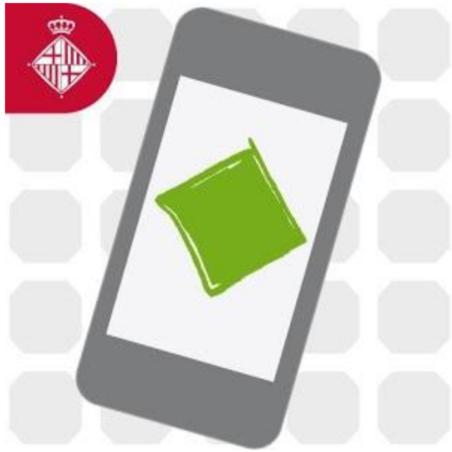

- Reduce traffic congestion
- Lower air and noise pollution

Icon of **apparkB** app for Android and iOS

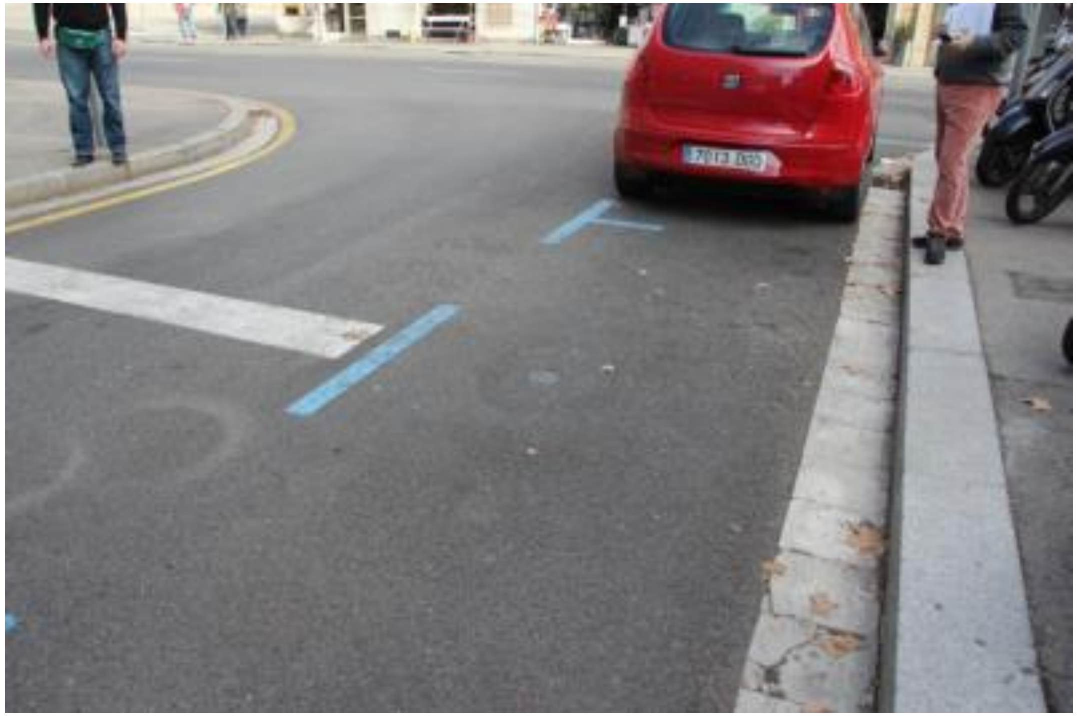

Image source: http://paulwallbank.com/2013/11/01/touring-barcelona-smart-city-internet-of-things/

Barcelona: an IoT-powered smart city

- A **city-wide network of sensors** provides real-time information on the flow of citizens, noise and other forms of environmental pollution, as well as traffic and weather conditions, enabling local authorities to streamline city operations for better environmental management, reduction of costs, and improvement of socioeconomic and environmental sustainability.
- Barcelona's highly-energy-efficient LED (Light-Emitting Diode) **streetlights** are fitted with CCTV (Closed-Circuit TeleVision), environmental monitoring sensors (humidity, temperature, air quality / pollution and noise) and WiFi.
- The streetlights are capable of dynamically managing the level of lighting depending on surrounding conditions to save energy, e.g., dim lights when no motion or pedestrians are detected in the street.

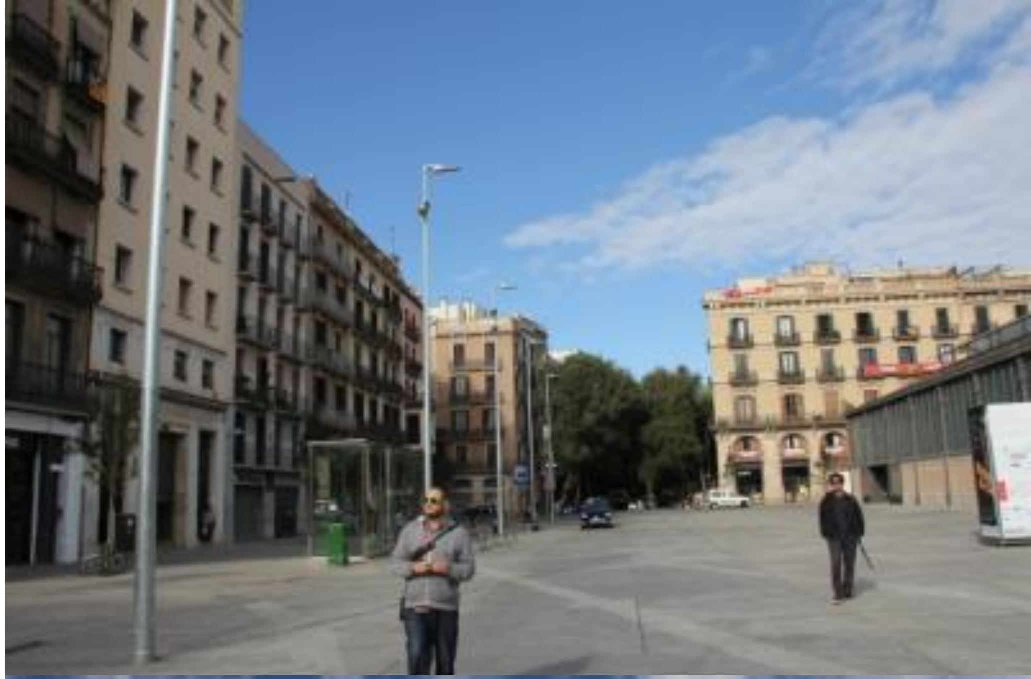

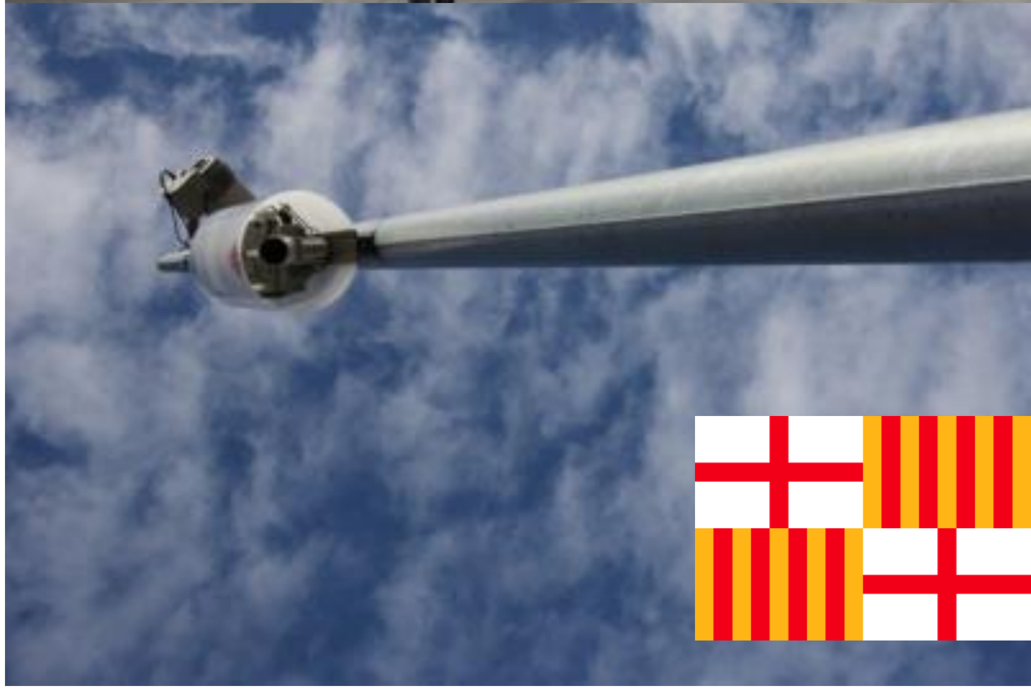

Image source: http://paulwallbank.com/2013/11/01/touring-barcelona-smart-city-internet-of-things/

Barcelona: an IoT-powered smart city

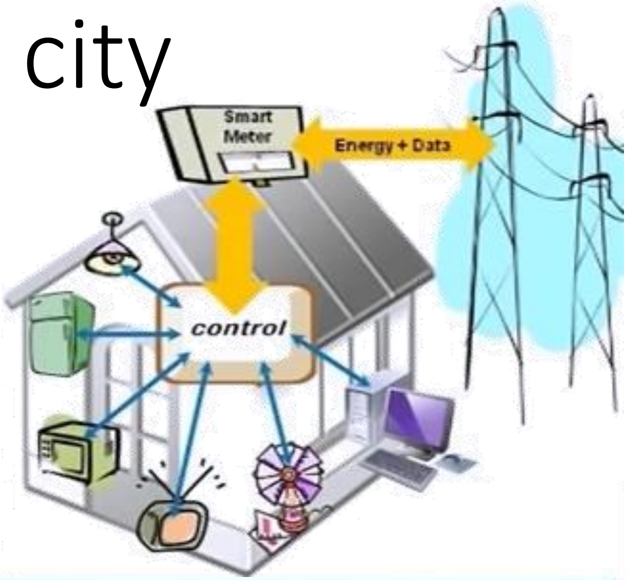

- Barcelona's **smart grid developments** include rolling out one million new smart electricity meters in the city, providing customers with higher energy usage awareness and optimisation tools, and helping them better plan and adapt their consumption for greater savings and reduction of greenhouse gas emissions such as CO_2 and NO_x.
- Barcelona is also a strong supporter of **electric vehicles** and created Spain's first electric vehicle fast recharge point.

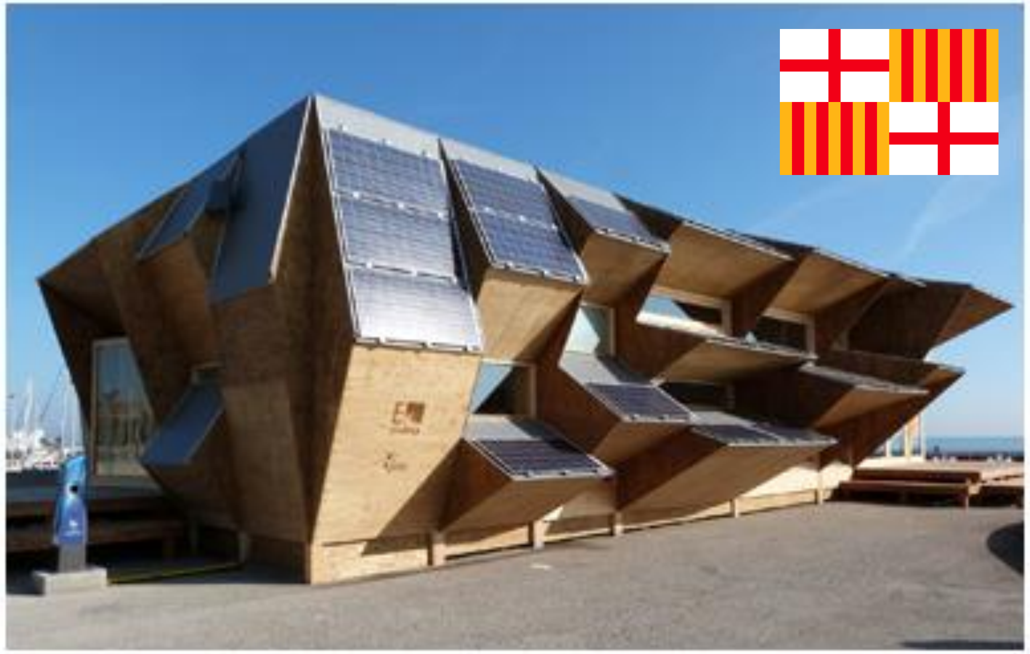

An innovative solar-powered building in Barcelona
Image sources: http://www.endesa.com/en/aboutEndesa/businessLines/principalesproyectos/Paginas/Barcelona_Smartcity.aspx and http://www.youtube.com/watch?v=QaTIt1C5R-M

Barcelona: an IoT-powered smart city

- Barcelona's wirelessly-connected **garbage bins** are fitted with sensors that monitor trash levels.
- The data reach the city council's team in charge, enabling the team to plan the optimal routes for garbage collection, update garbage truck drivers in real time regarding which routes to take, and in this way optimise productivity and reduce waste management service costs, as well as pollution caused by garbage trucks.
- Other compact drop-off street containers connect through pipes to a subterranean vacuum network sucking up trash below the ground, thus decreasing noise and air pollution caused by garbage trucks.

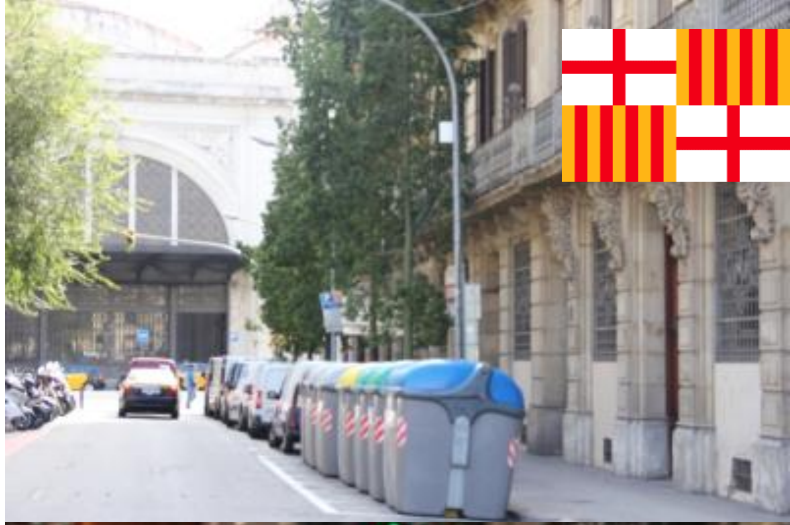

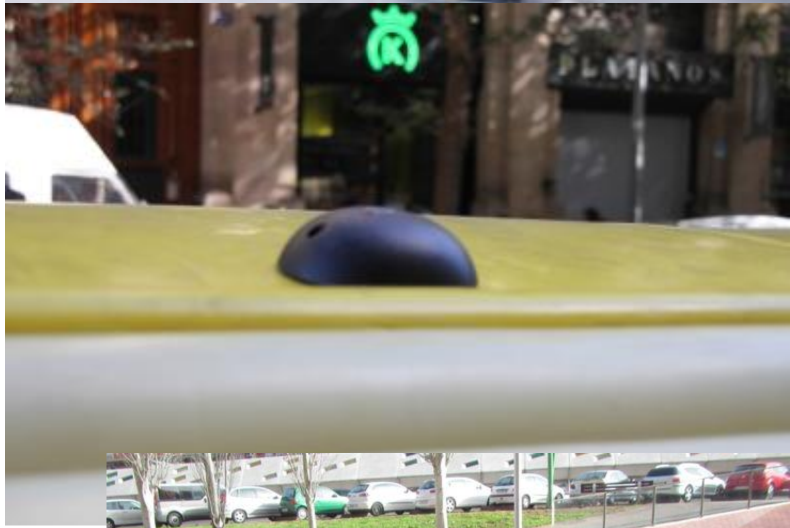

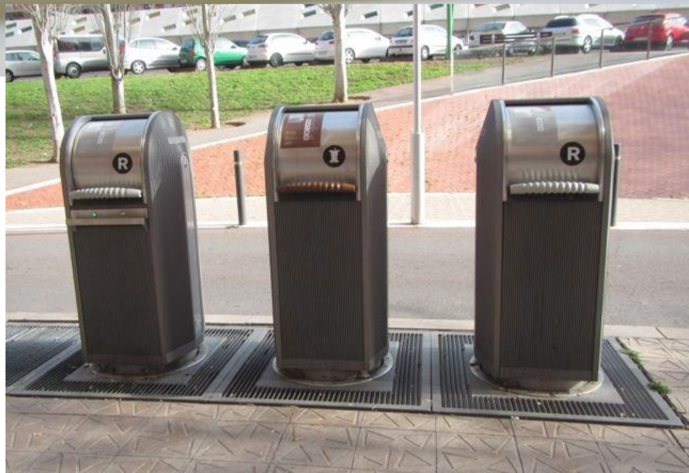

Image sources: http://paulwallbank.com/2013/11/01/touring-barcelona-smart-city-internet-of-things/ and http://www.vilaweb.cat/noticia/4175829/20140226/ten-reasons-why-barcelona-is-smart-city.html

Barcelona: an IoT-powered smart city

- **Smart parks** at Sant Cugat (a suburb 20 Km north of Barcelona) are watered only when necessary, thanks to sensors capturing the degree of soil humidity and air temperature in order to determine the exact amounts of water needed.

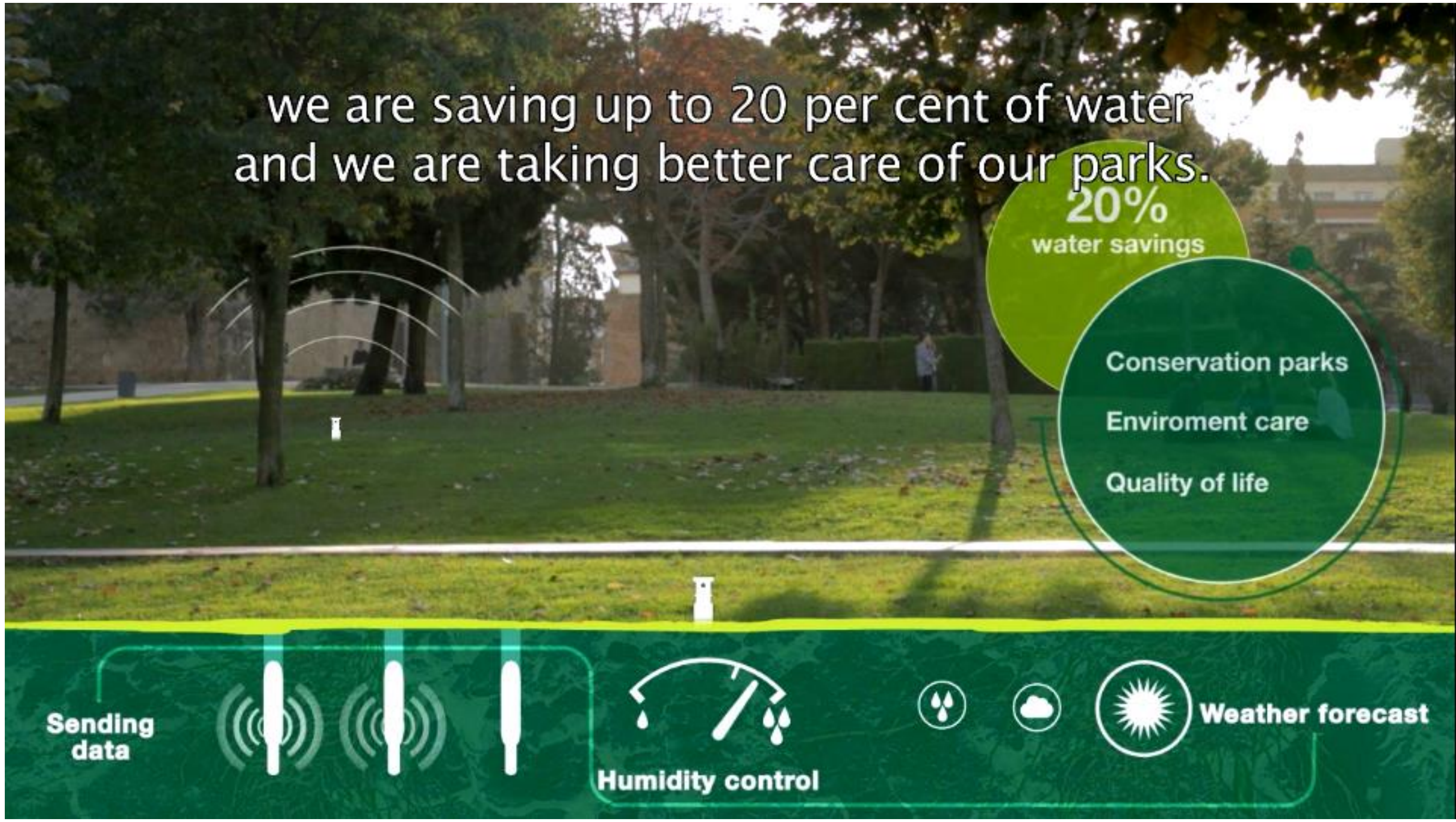

Image source: http://vimeo.com/32661217

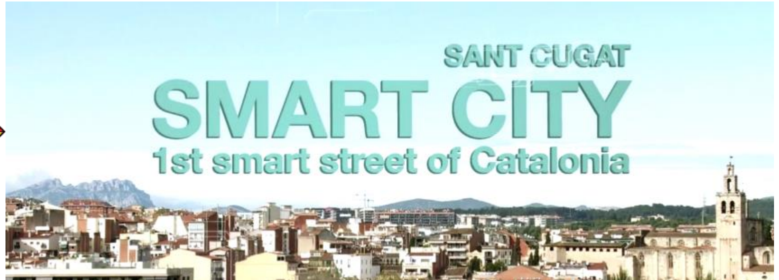

Video: Sant Cugat (a suburb of Barcelona) smart city pilot

- Length: 04:05 - http://vimeo.com/32661217 (also locally embedded on next slide)

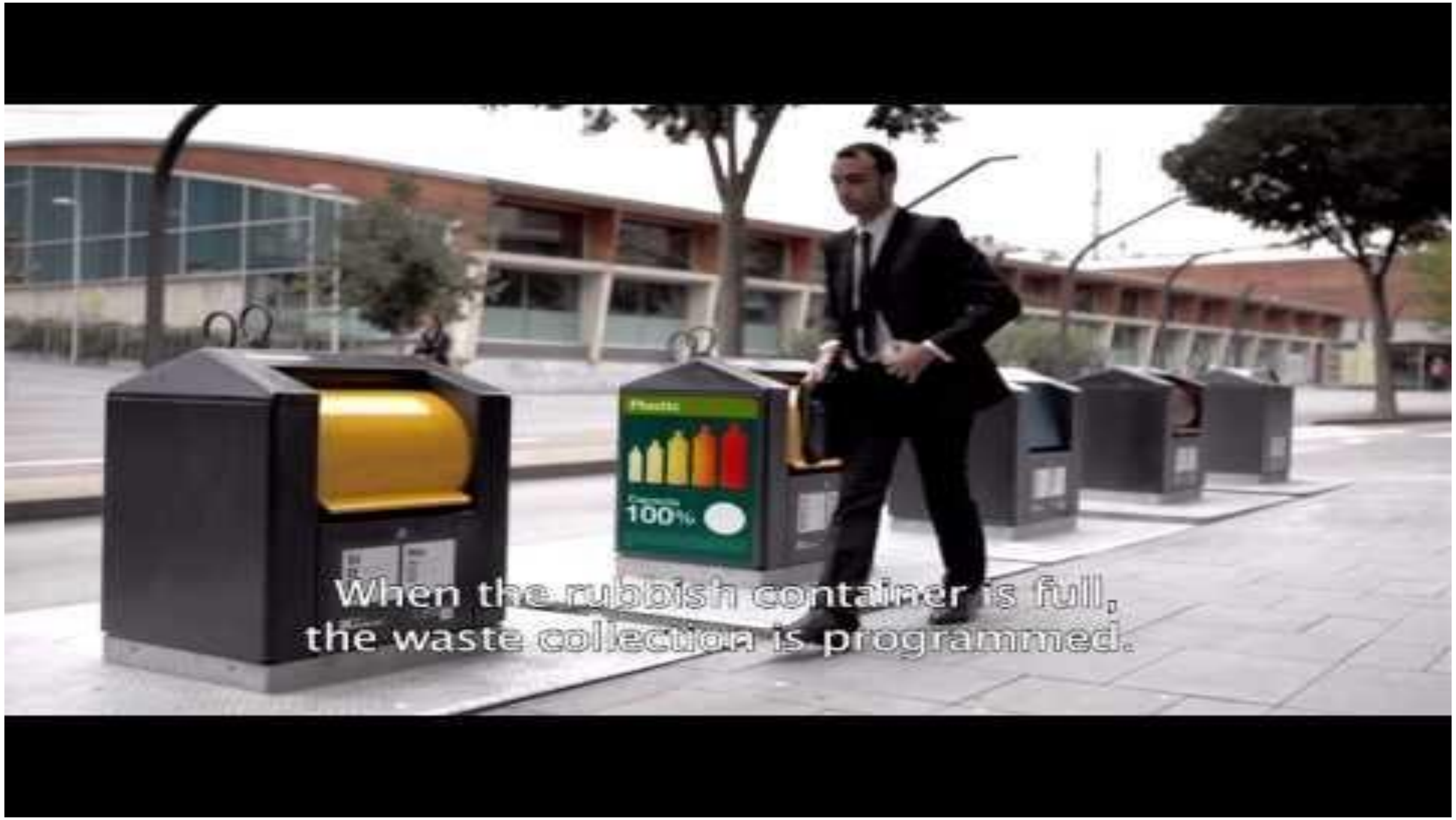

See also:
Barcelona embraces IoT to create a smart city (03:50) - http://www.youtube.com/watch?v=TCbvxb5t5_8

Embedded video is not available in this copy of the presentation. Please refer to the previous slide for the YouTube version.

Barcelona: benefits of being an IoT-powered smart city

- **An economic growth opportunity:** Thanks to IoT, the city was able to make big savings in areas such as smart water (savings of €42.5 million a year) and lighting and parking management (increased revenues of parking fees by 33% or €36.5 million), besides creating 47,000 new jobs related to the smart city developments in Barcelona (source: http://www.forbes.com/sites/connieguglielmo/2014/01/07/ces-live-cisco-ceo-chambers-to-deliver-keynote/).

- **Better quality of life for citizens and visitors:** According to Antoni Vives, Barcelona's Deputy Mayor for Urban Habitat, the main rationale behind his city's embracement of IoT is to improve the quality of life of people. Antoni Vives describes his ultimate vision of a smarter Barcelona by 2023 as "*a city of culture, creativity, knowledge but mainly fairness and well-being; a place where people live near where they work; a city self-sufficient in energy; a zero emission city and a city hyperconnected to the world*".

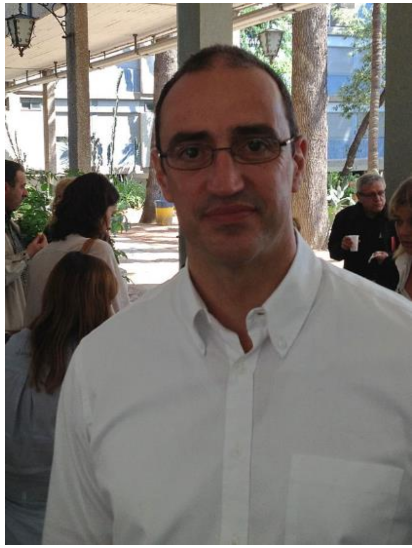

There are many more smart city examples around the world

- Video: **Nice**, France: an IoT-powered smart city
  Length: 3:41 -
  https://www.youtube.com/watch?v=neVyOTXB4eI
- Video: **Hamburg**, Germany: an IoT-powered smart city
  Length: 3:28 -
  https://www.youtube.com/watch?v=FoEPlE8Pg7I
- Video: **Milton Keynes**, England, UK - MK:Smart
  Length: 2:58 -
  https://www.youtube.com/watch?v=6KIu3ojtrNs
- And many more cities…
- Related video: Internet of Things World Barcelona 2013 Keynote Demo: **additional creative IoT application examples for cities** (CISCO)
  Length: 13:31 –

  http://www.youtube.com/watch?v=IpRrYbHiKAM

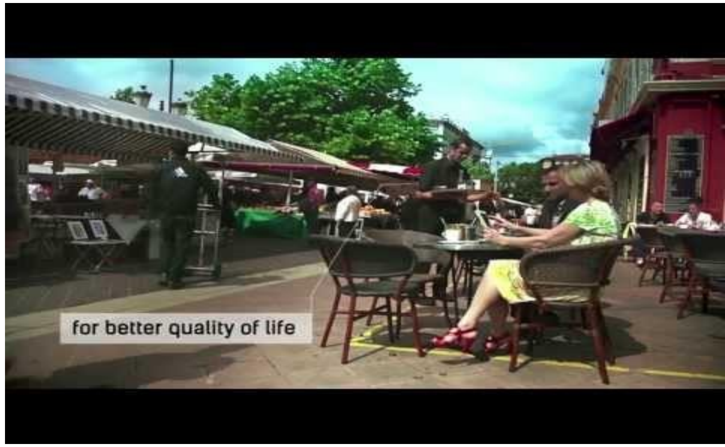

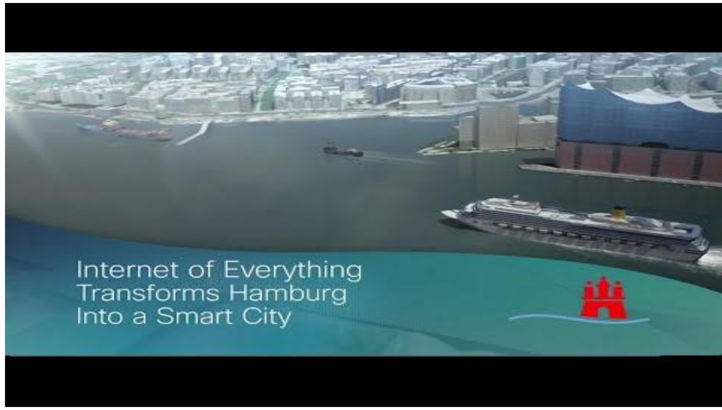

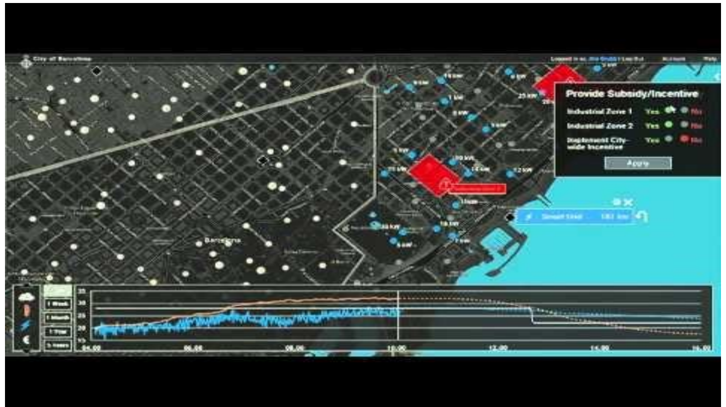

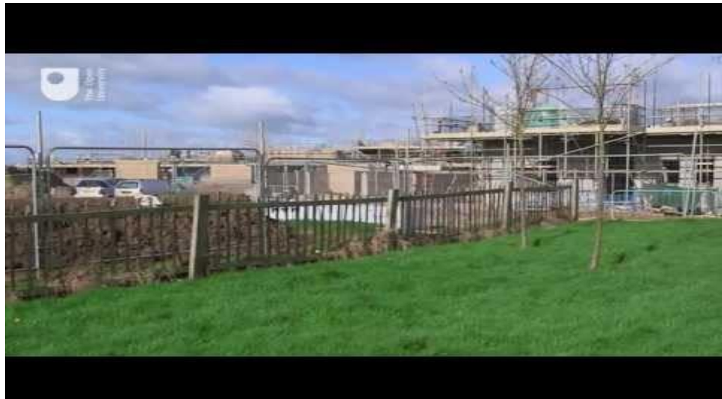

IoT data and device privacy and security

- As more of our personal 'quantified self' (health and other) data, and data originating from our devices, appliances, vehicles, offices and homes get stored, processed, interlinked and shared online on the 'cloud', questions and concerns arise about data privacy, misuse (e.g., unauthorised sharing / unwanted advertising) and security / hacking attacks.

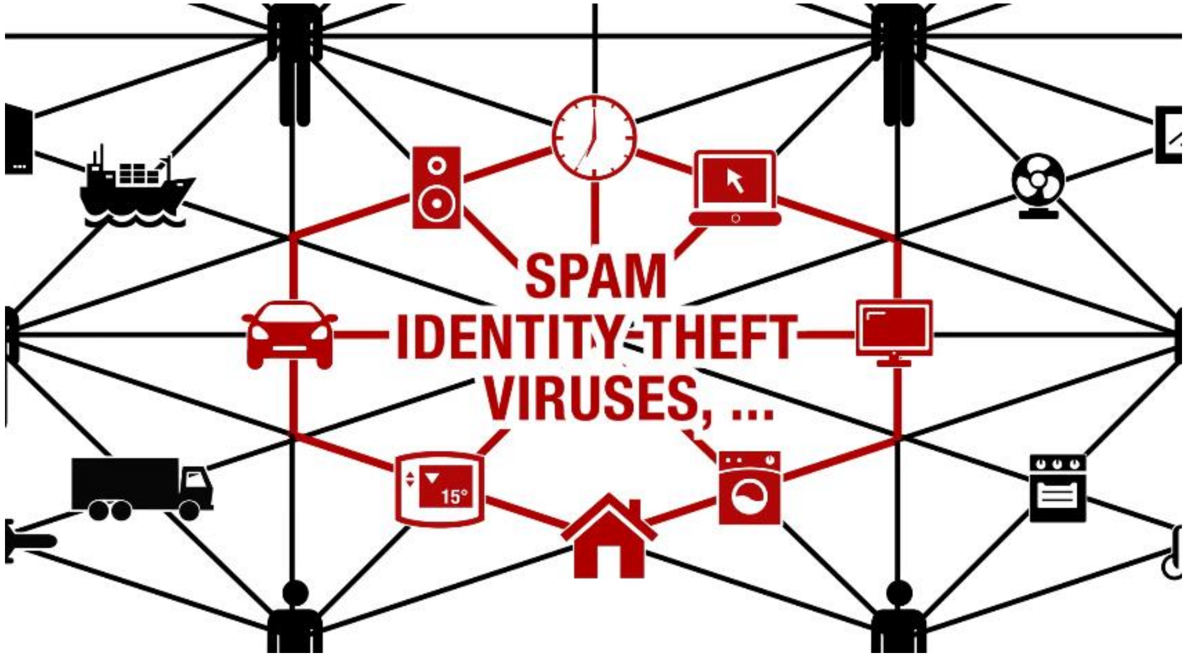

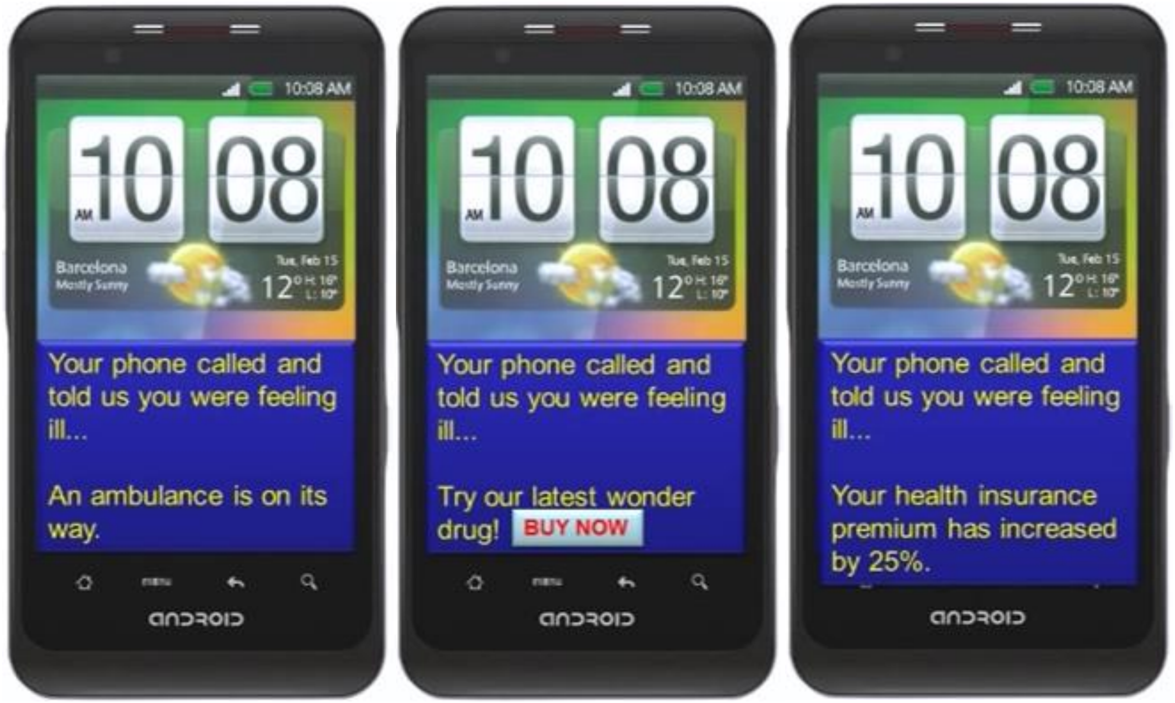

Image sources: https://www.youtube.com/watch?v=qfhcPvwzJF0 and https://www.youtube.com/watch?v=QaTIt1C5R-M (Dr John Barrett, Cork Institute of Technology)

IoT data and device privacy and security

BBC NEWS TECHNOLOGY

Home | World | UK | England | N. Ireland | Scotland | Wales | Business | Politics | Health | Education | Sc

21 November 2013 Last updated at 17:49

Share

LG promises update for 'spying' smart TV

By Jane Wakefield
Technology reporter

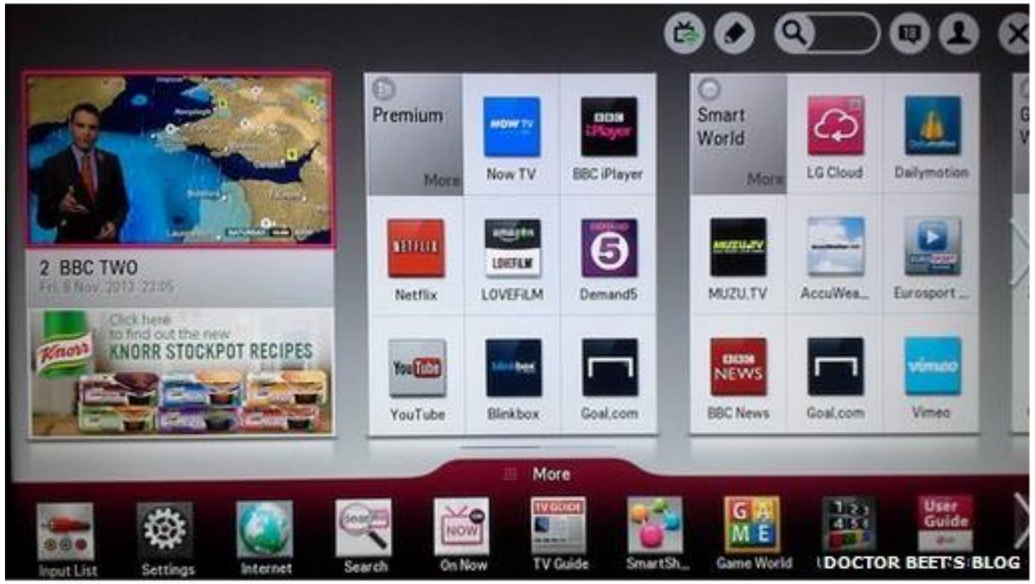

Mr Huntley wanted to know how LG determined which ads should be shown on his TV

LG has admitted it continued collecting data on viewing habits even after users had activated a privacy setting designed to prevent it.

Related Stories

http://www.bbc.co.uk/news/technology-25042563

Is your smart TV SPYING on your family? Investigation reveals how much personal data television sets know about viewers

- Which? experts monitored streams of data coming from 2013 and 2014 models of smart TVs from LG, Samsung, Sony, Panasonic and Toshiba
- They discovered all the brands track people's viewing habits to some extent
- They are given permission to do this in the TV's terms and conditions
- If viewers reject conditions they can't access some features on their TV sets - and Samsung and Toshiba owners can't use them at all

By SARAH GRIFFITHS FOR MAILONLINE

PUBLISHED: 14:55, 29 August 2014 | **UPDATED:** 12:04, 1 September 2014

"Smart TV brands monitor the programmes you watch and the websites you browse to offer personalised recommendations for TV shows - but this information can be passed on to advertisers to target viewers more accurately"

http://www.dailymail.co.uk/sciencetech/article-2737708/Is-smart-TV-SPYING-family-Investigation-reveals-personal-data-TV-sets-knows-you.html

Video excerpts from 'BBC Horizon 2014-2015 Episode 4: Inside the Dark Web'

- Length: 6:06 - http://www.bbc.co.uk/programmes/p025z1kq (also locally embedded on next slide)

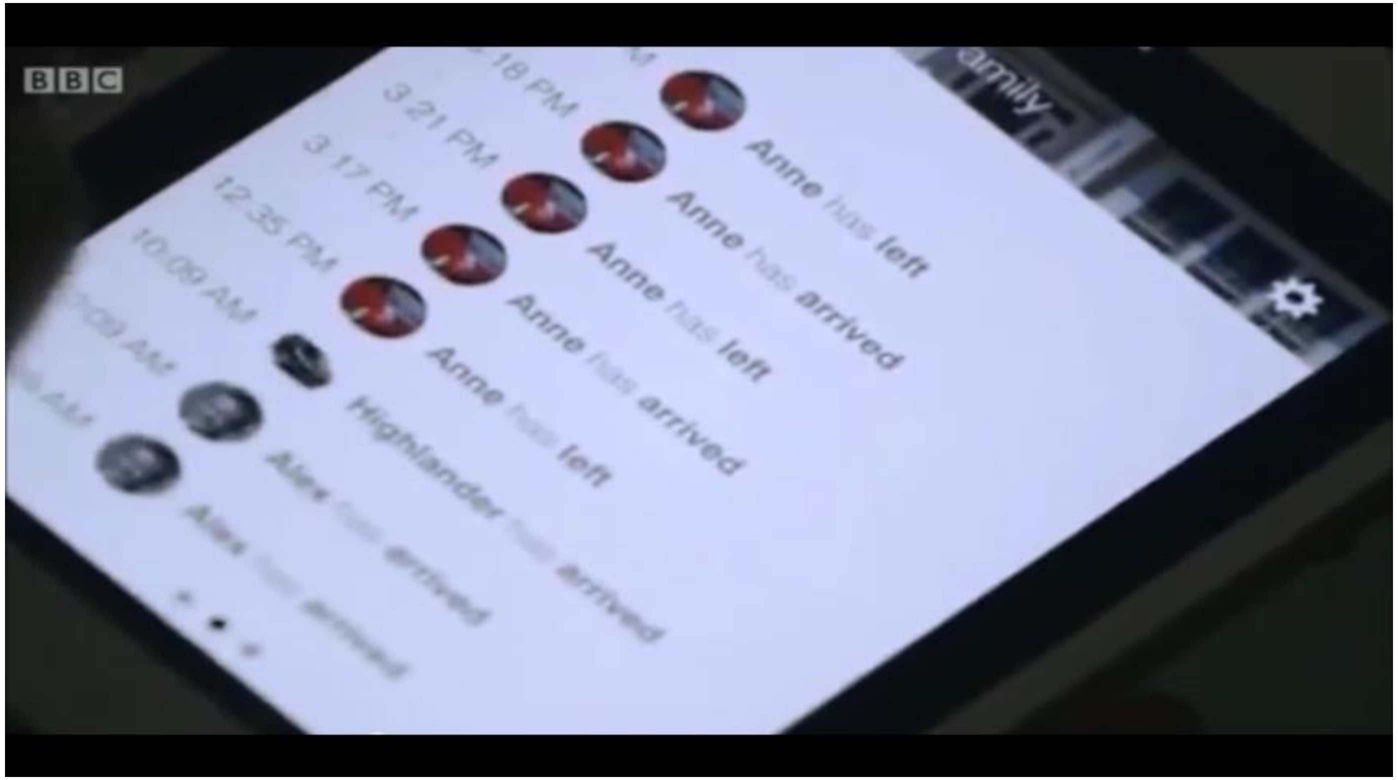

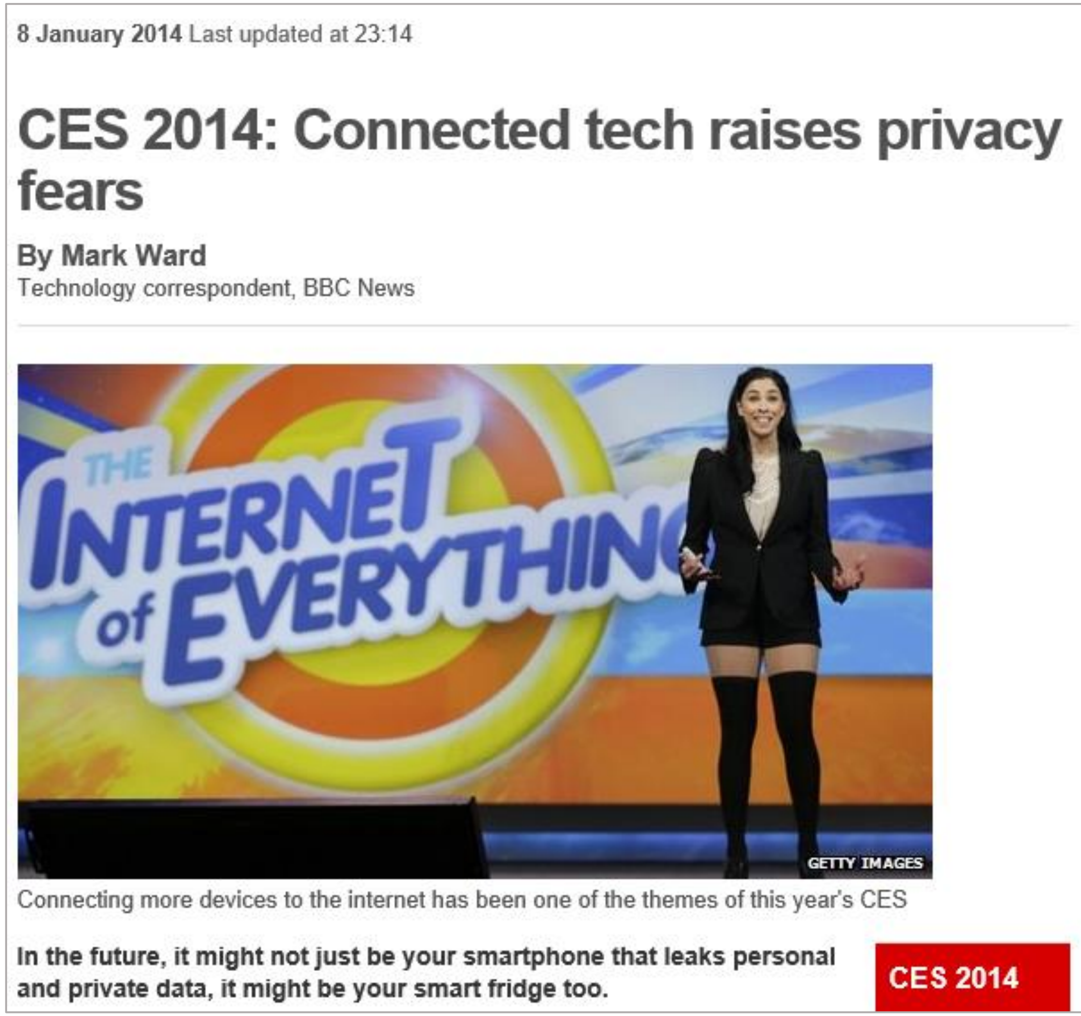

8 January 2014 Last updated at 23:14

CES 2014: Connected tech raises privacy fears

By Mark Ward
Technology correspondent, BBC News

Connecting more devices to the internet has been one of the themes of this year's CES

In the future, it might not just be your smartphone that leaks personal and private data, it might be your smart fridge too.

CES 2014

Image source: http://www.bbc.co.uk/news/technology-25662006

Embedded video is not available in this copy of the presentation. Please refer to the previous slide for the YouTube version.

IoT data and device privacy and security

- **Data ownership and 'right to be forgotten' issues**: "*[Consumers] may think we're in charge of our shopper cards and our mobile apps and our smart fridges—but … let's not fool ourselves. [The information] is not ours. It belongs to Google, and IBM, and Cisco Systems…and the global Mega-Corp that owns your local supermarket. If you don't believe us, just try removing 'your' data from their databases.*"
  --Katherine Albrecht and Katina Michael (2013)

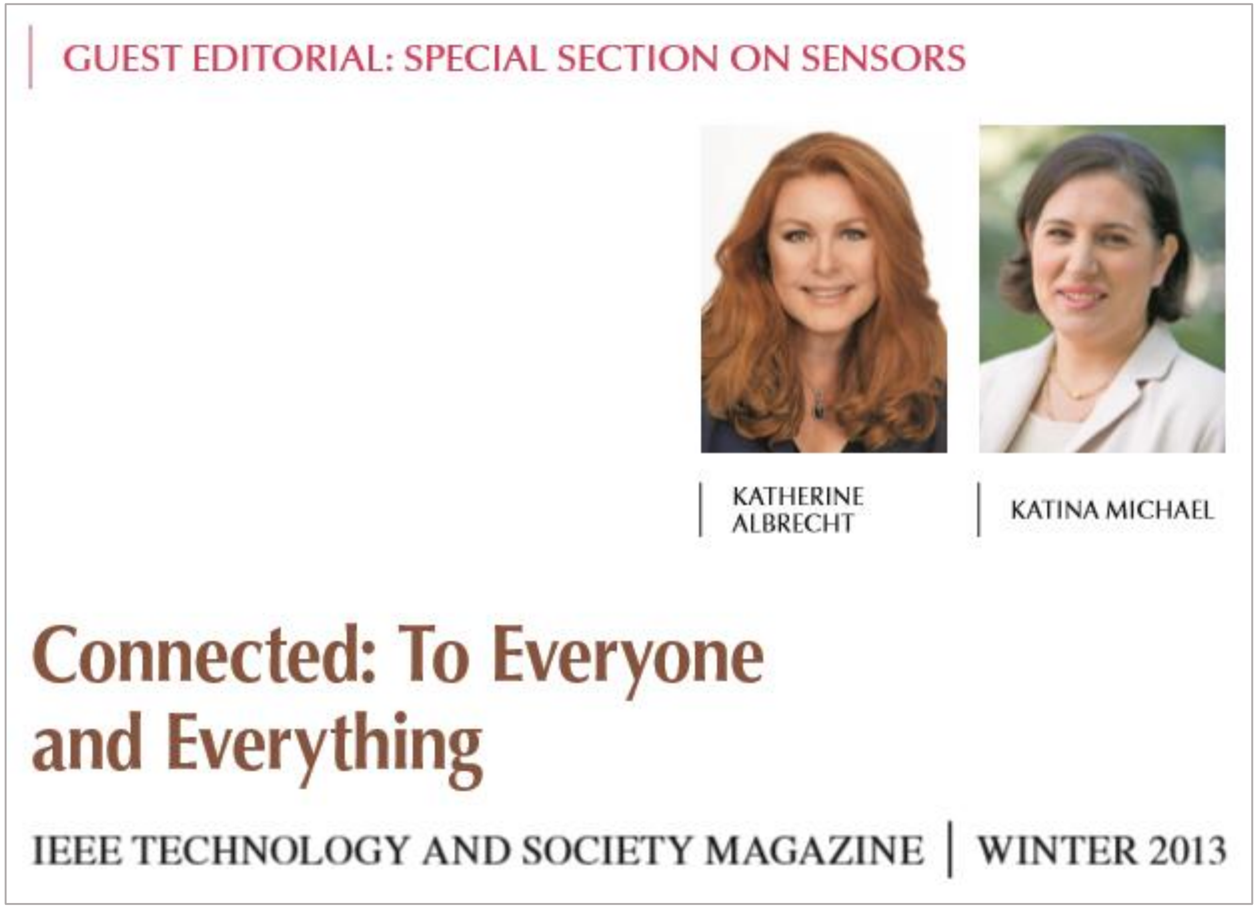

Image source: http://ieeexplore.ieee.org/xpl/articleDetails.jsp?arnumber=6679311

IoT data and device privacy and security

Scientists Demonstrate Deadly WiFi Pacemaker Hack

http://www.wired.com/2008/03/scientists-demo/

Insulin pump hack delivers fatal dosage over the air

Attack code for 'unpatchable' USB flaw released

http://www.bbc.co.uk/news/technology-29475566

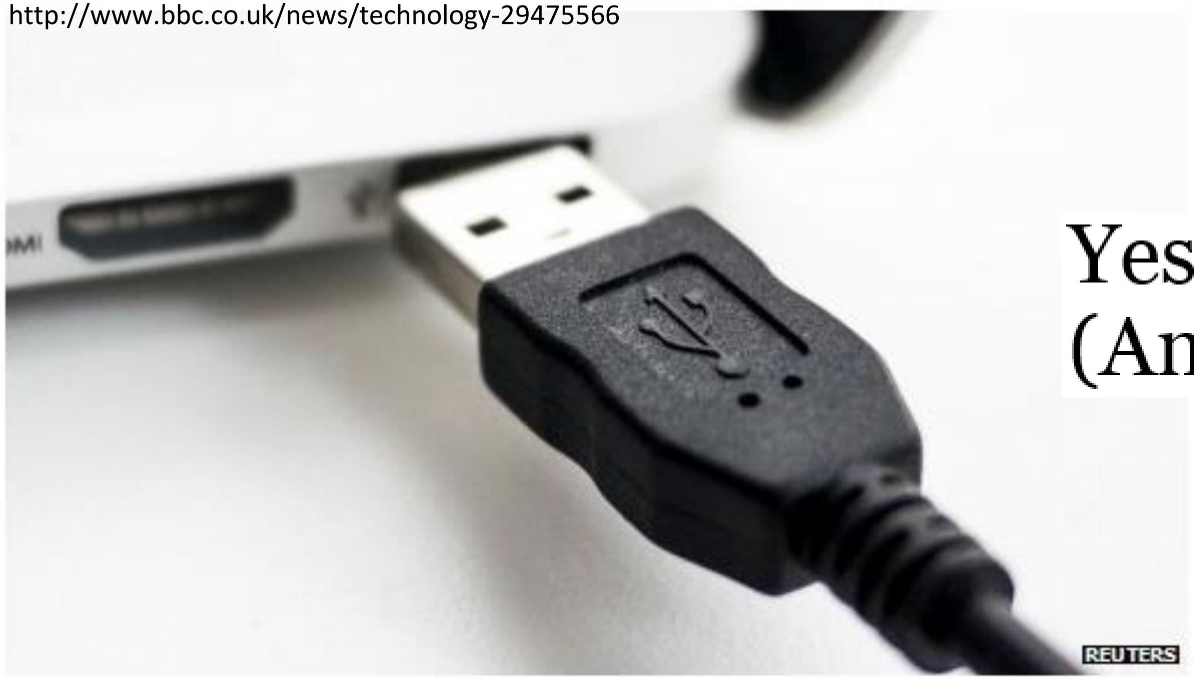

The USB flaw means attackers can implant code on almost any USB-using device

http://krebsonsecurity.com/2012/04/fbi-smart-meter-hacks-likely-to-spread/

Researchers Show How a Car's Electronics Can Be Taken Over Remotely

http://www.nytimes.com/2011/03/10/business/10hack.html

Yes, You Can Hack A Pacemaker (And Other Medical Devices Too)

http://www.forbes.com/sites/singularity/2012/12/06/yes-you-can-hack-a-pacemaker-and-other-medical-devices-too/

http://krebsonsecurity.com/2012/04/fbi-smart-meter-hacks-likely-to-spread/

FBI: Smart Meter Hacks Likely to Spread

IoT data and device privacy and security: some possible solutions

- Individuals can be offered an option to **'opt out'** of syncing their data with third-party or public cloud databases and services **and become their own service providers**. Using home (private) cloud-computing systems, individuals store their information privately instead of relying on companies, and choose what data leave their homes (or private cloud) and become part of the public cloud and what do not. However, by doing so, they might lose some features and functionality offered by third-party processing and interlinking* of their data (see http://theinstitute.ieee.org/technology-focus/technology-topic/the-value-of-privacy).
- In its report "*IoT Privacy, Data Protection, Information Security*" published in January 2013, the European Union recommends the development of **privacy-friendly default settings on IoT products and services** that would give users more control over what information is shared with others. It also suggests that IoT networks **give individuals the rights to their own data**.

---

* with additional own data and/or others' data, e.g., to answer the question: 'how do I compare to others'.

European Commission
DIGITAL AGENDA FOR EUROPE
A Europe 2020 Initiative

Conclusions

- IoT-powered smart cities aim at **improving the quality of life** of their populations in a variety of ways, including through measures that promote **eco-friendly, sustainable environments** and the delivery of **'connected health/care' services** to citizens at home and on the move.
- Smart cities stand better chances of becoming **healthier cities**. The WHO and associated national Healthy Cities networks have hundreds of member cities around the world that could benefit from, and harness the power of, IoT to improve the health and well-being of their local populations.
- IoT-powered smart cities rely on a growing number of sub-technologies and subsystems that need to be seamlessly interconnected and interfaced with one another in real time. This can only be achieved through the adoption of adequate **standards and protocols** for measurement, communication, integration, interoperability and control.

Conclusions

- IoT sensors, devices, systems and services generate big amounts of **real-time** data, much of which are **geo-located**. The main challenge facing smart cities and smart-cities-to-be is how to make sense and best use of such '**big data**', while preserving citizens' **privacy** and **data security**. Cities can only be really 'smart' if they have in place the necessary intelligent, robust and reliable **analytics** functions to integrate and synthesise these big data, and filter out any unwanted 'noise' for the purposes of better planning and managing city operations and systems (including healthcare).
- **Innovation** is key to avoiding collapse and **promoting sustainability** of large cities and their infrastructures. IoT for cities is a major innovation, able to stimulate other forms of innovation, and in doing so, **generate economic value, growth and competitive advantage**.

The fourth industrial revolution is here!

- "*The Internet of Things represents the fourth industrial revolution, opening up opportunities for a more enjoyable life and new and better ways of doing business. While IoT will create entirely new business models, innovations will have to come more quickly for companies to stay relevant. To do this, managers need to envision the valuable new opportunities that become possible when the physical world is merged with the virtual world and where potentially every physical object can be both intelligent and networked. Starting now, they must create the organisations and IoT-based business models that can turn these ideas into reality.*"

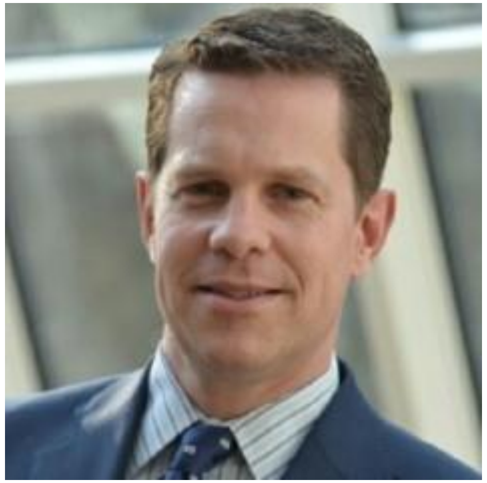

--**Matthew Jennings**, Managing Director, Bosch Software Innovations (2014)

Third Industrial Revolution (beginning from the late 1950s to the late 1970s): the change from analogue, mechanical and electronic technology to digital technology (start of the 'Information Age').
Second Industrial Revolution (2nd half of the 19th century until World War I): the period from the introduction of Bessemer steel in the 1860s to early factory electrification, mass production and the production line (Ford Model T car in 1908).
First Industrial Revolution (2nd half of the 18th century until 1820-1840): the transition to new manufacturing processes, going from hand production methods to machines, with new chemical manufacturing and iron production processes, improved efficiency of water power, increasing use of steam power and the change from wood fuel to coal.

INTERNATIONAL JOURNAL OF HEALTH GEOGRAPHICS

Founder & Editor-in-Chief:
Maged N. Kamel Boulos (UK)

IMPACT FACTOR **1.97**

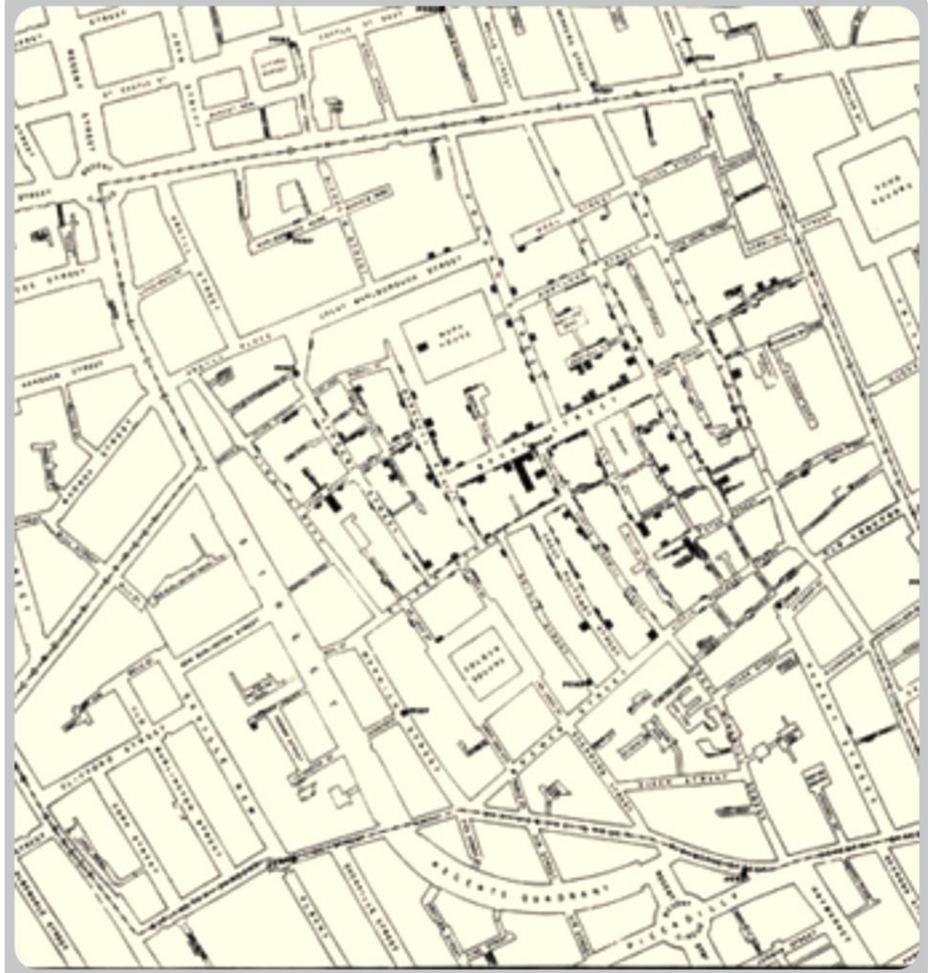

- **2013 Impact Factor 1.97**
- **2013 5-Year Impact Factor 2.675**
- **High-quality content**
- **Covers a wide range of interdisciplinary geospatial topics**

SpringerLink FULL-TEXT ARTICLE | OPEN ACCESS | UK PUBMED CENTRAL | NLM PubMed.gov | PubMed Central | THOMSON REUTERS | MEDLINE

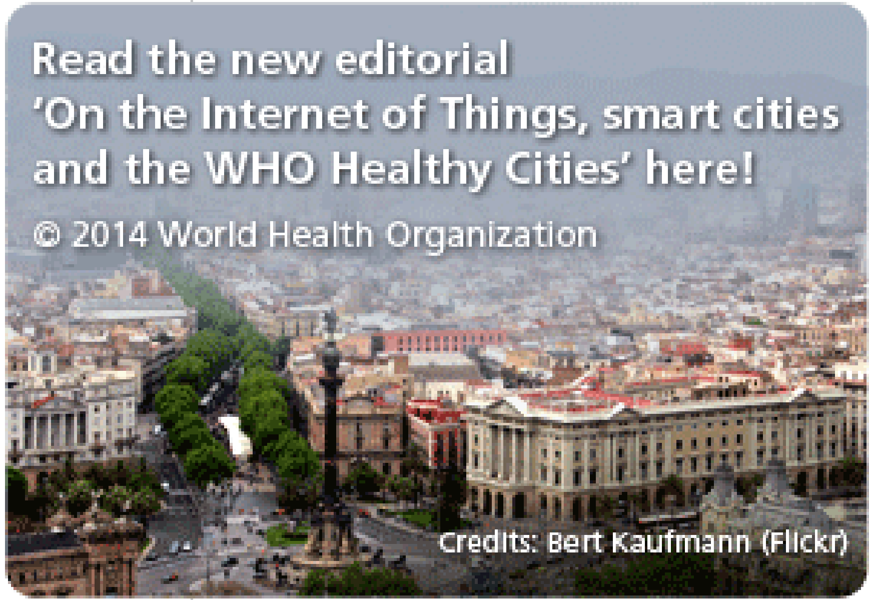

http://www.ij-healthgeographics.com/content/13/1/10

Springer

BioMed Central
The Open Access Publisher
www.biomedcentral.com

For discussion with participants:

- Smart cities as **greener**, **more sustainable** cities.
- Smart cities for **active ageing**.
- **Privacy and security** concerns in IoT / smart cities.
- The need for **high speed Internet infrastructure** (*cf.* Barcelona's 500 Km fibre network and in UK, the government's investment of £146m in broadband in the Highlands and Islands, Scotland, and similar investments in SW England).
- Smaller towns and rural areas should not be left behind: the emerging concept of '**distributed** cities', e.g., in the Scottish Highlands.
- Issues of usability, **accessibility** (including cognitive accessibility / **literacy** issues) and **inclusion** / **equity**.
- **Citizen engagement and participation**, e.g., citizen sensing.
- **Focus on, and engage, People. Avoid dehumanising** approaches and terminology. People are very much more than just 'connected things'; say (and practise) 'Internet of Things *and People*'.

Concepts map (by MN Kamel Boulos):

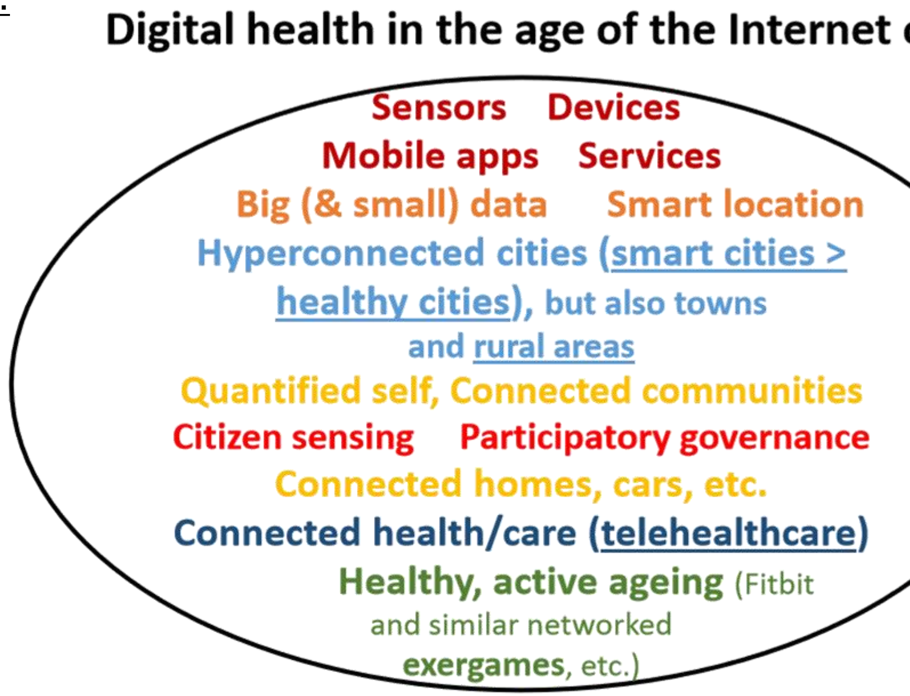

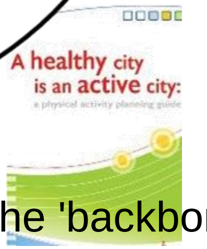

Background material:

Mapping smart cities in the EU

http://www.europarl.europa.eu/RegData/etudes/etudes/join/2014/507480/IPOL-ITRE_ET(2014)507480_EN.pdf

Discussant concluding thoughts: IoT represents the fourth industrial revolution and is the 'backbone' of smart cities. Smart cities stand better chances of becoming healthier cities.
